# Assessing the Effects of Climate Change and Air Pollution on Soil Properties and Plant Diversity in Northeastern U.S. Hardwood Forests: Model Setup and Evaluation

**DOI:** 10.1007/s11270-019-4145-6

**Published:** 2019-04-29

**Authors:** Salim Belyazid, Jennifer Phelan, Bengt Nihlgård, Harald Sverdrup, Charles Driscoll, Ivan Fernandez, Julian Aherne, Leslie M. Teeling-Adams, Scott Bailey, Matt Arsenault, Natalie Cleavitt, Brett Engstrom, Robin Dennis, Dan Sperduto, David Werier, Christopher Clark

**Affiliations:** Department of Physical Geography, Stockholm University, Stockholm, Sweden; RTI International, Raleigh, NC, USA; Department of Biology, Lund University, Lund, Sweden; Department of Industrial Engineering, University of Iceland, Reykjavik, Iceland; Department of Civil and Environmental Engineering, Syracuse University, Syracuse, NY 13244, USA; School of Forest Resources and Climate Change Institute, University of Maine, Orono, ME, USA; Trent University, Trent, Canada; Department of Life and Physical Sciences, Great Bay Community College, Portsmouth, NH, USA; USDA Forest Service, Northeastern Research Station, Campton, NH, USA; Stantec Consulting Services, Inc., Topsham, ME, USA; Department of Natural Resources, Cornell University, Ithaca, NY 14853, USA; Counsulting Botanist and Ecologist, Marshfield, VT, USA; National Exposure Research Laboratory, Environmental Protection Agency, Triangle Park, NC 27711, USA; USDA Forest Service, White Mountain National Forest, 71 White Mountain Dr., Campton, NH 03223, USA; David Werier Botanical and Ecological Consulting, Willseyville, NY, USA; U.S. Environmental Protection Agency, Washington, DC, USA

**Keywords:** Ecosystem modeling, Hubbard Brook, Bear Brook, ForSAFE-Veg, Vegetation modeling, Plant biodiversity

## Abstract

The integrated forest ecosystem model ForSAFE-Veg was used to simulate soil processes and understory vegetation composition at three—sugar maple, beech, yellow birch—hardwood forest sites in the Northeastern United States (one at Hubbard Brook, NH, and two at Bear Brook, ME). Input data were pooled from a variety of sources and proved coherent and consistent. While the biogeochemical component ForSAFE was used with limited calibration, the ground vegetation composition module Veg was calibrated to field relevés. Evaluating different simulated ecosystem indicators (soil solution chemistry, tree biomass, ground vegetation composition) showed that the model performed comparably well regardless of the site’s soil condition, climate, and amounts of nitrogen (N) and sulfur (S) deposition, with the exception of failing to capture tree biomass decline at Hubbard Brook. The model performed better when compared with annual observation than monthly data. The results support the assumption that the biogeochemical model ForSAFE can be used with limited calibration and provide reasonable confidence, while the vegetation community composition module Veg requires calibration if the individual plant species are of interest. The study welcomes recent advances in empirically explaining the responses of hardwood forests to nutrient imbalances and points to the need for more research.

## 1 Introduction

Atmospheric deposition of nitrogen (N) and sulfur (S) and climate change are two of the most prominent global change drivers induced by human activity (Rockström et al. 2009). Together, the individual and interactive effects from these two global change factors influence many aspects of ecosystem structure and function, including plant community diversity and composition, as well as soil processes such as carbon (C) sequestration and nutrient cycling (Porter et al. 2013).

Nitrogen limits the productivity of many ecosystems (Aerts and Chapin 2000; Aber et al. 1998; Vitousek et al. 1997; Vitousek and Howarth 1991), and human population growth and industries have increased N deposition in many regions by nearly an order of magnitude over historic levels (Galloway et al. 2004; Vitousek et al. 1997). Increased N deposition leading to eutrophication can cause a decline in species richness or evenness in vulnerable ecosystems (Pardo et al. 2011; Bobbink et al. 2010; Bowman et al. 2010; Emmett 2007; McDonnell et al. 2018a), and declines in tree growth and increases in mortality have also been noted (Thomas et al. 2010; Wallace et al. 2007). Once N availability exceeds combined plant and microbial demands, terrestrial ecosystems can become N saturated, resulting in nitrate (NO_3_^−^) leaching from soils to nearby aquatic systems (Emmett 2007; Aber et al. 1998; Aber, and P.,, and Melillo, J.M. 1989; Stoddard 1994). In addition to eutrophying effects, N and S deposition can also have acidifying effects on terrestrial ecosystems through damage to foliage and altered soil chemistry (Bobbink et al. 2010; Bobbink and Hettelingh 2011; Pardo et al. 2011). Lesions, chlorosis, and necrosis can develop in foliage, base cations can leach from the soil and cause nutrient imbalances, and reductions in soil pH may increase the solubility of phytotoxic aluminum (Al) (Bobbink et al. 2010). Plants vary in their sensitivity to the edaphic stresses created by elevated N and S deposition (Cronan and Grigal 1995; Sverdrup and Warfvinge 1993), which can lead to changes in plant species composition.

Climate change impacts plants communities and soil processes. Changes in air temperature and precipitation patterns can influence tree and plant productivity, distribution and ranges, and phenology (McNulty and Boggs, McNulty and Boggs 2010; Thuiller et al. 2008; Parmesan 2006), ultimately impacting species composition and biodiversity in terrestrial ecosystems (Porter et al. 2013). Higher temperatures can result in longer growing seasons and contribute to species migrations to higher latitudes and altitudes (Parmesan 2006). Increased air temperatures can increase evapotranspiration rates and water stress (McDonnell et al. 2013), and altered precipitation patterns can result in drought in some locations and increased water availability in others (Parmesan 2006).

Changes in climate and deposition can also be expected to interactively impact terrestrial diversity and soil processes. Responses may be additive, synergistic, and/or antagonistic depending on the ecosystem and conditions (Shaw et al. 2002; Zavaleta et al. 2003; Dukes et al. 2005). For example, species diversity is typically decreased by increased N deposition, but reductions in diversity can be offset with elevated CO_2_ concentrations (Reich 2009). Similarly, increased spring temperatures may allow forest species to begin growing earlier in the season, but increased N deposition may increase the sensitivity of species to frost and drought (McNulty and Boggs 2010; Porter et al. 2013). With respect to soils, studies have reported stimulation of mineralization with N additions that is further enhanced with elevated CO_2_ and precipitation (Niboyet et al. 2011). Similar results were reported for the influences of N additions and soil warming on nitrification (Ma et al. 2011).

The objective of this study was to test the ability of a dynamic modeling framework, ForSAFE-Veg (Sverdrup and Belyazid 2014), to evaluate the interactive impacts of deposition and climate change on soil processes and understory plant diversity in hardwood forests in the Northeastern United States (U.S.). This study presents the model setup and evaluation used as the basis of a further study documented in Phelan et al. (2016) for assessing the combined effects of climate change and air pollution on soil properties and plant diversity at the experimental sites described here.

## 2 Method

### 2.1 Model and Site Selection

Prior to this study, a set of models were reviewed to support the study goals, including six biogeochemical models (DayCent, DayCent-Chem, PnET-BGC, MAGIC, ForSAFE, VSD) and one plant community dynamics model (Veg) (RTI 2012) (see Cosby et al. (1985), Alveteg et al. 1998a, [Bibr R7][Bibr R69], Gbondo-Tugbawa et al. (2001), Hartman et al. (2007), Posch and Reinds (2009) and Belyazid et al. (2011b) for more information about the individual models). Although other vegetation response models including GLOBIO/IMAGE, SORTIE, ED, BERN, and MOVE/NTM have been recently reviewed and were initially considered (de Vries et al. 2010), only Veg was reviewed in detail because it is able to simulate responses at the individual plant-species level and/or is not restricted to European plant species (Sverdrup et al. 2012; McDonnell et al. 2014). ForSAFE was selected for this study because the model (a) has been previously used to evaluate the impacts of deposition and climate change on nutrient cycling and critical loads of atmospheric N and S deposition (Posch et al. 2011; Zanchi et al. 2014; Belyazid et al. 2011a, 2011b; Sverdrup and Belyazid 2014), (b) is able to simulate soil base cation weathering (BCw), biogeochemical indicators of critical loads, and all parameters required for vegetation response modeling (Veg), and (c) is fully-integrated with Veg. Combined, ForSAFE-Veg represents a model chain of dynamic biogeochemistry (ForSAFE, Wallman et al. 2005) and plant responses (Veg, Belyazid 2006) (see RTI (2012) for a more detailed description of the models review).

**Fig. 1:**
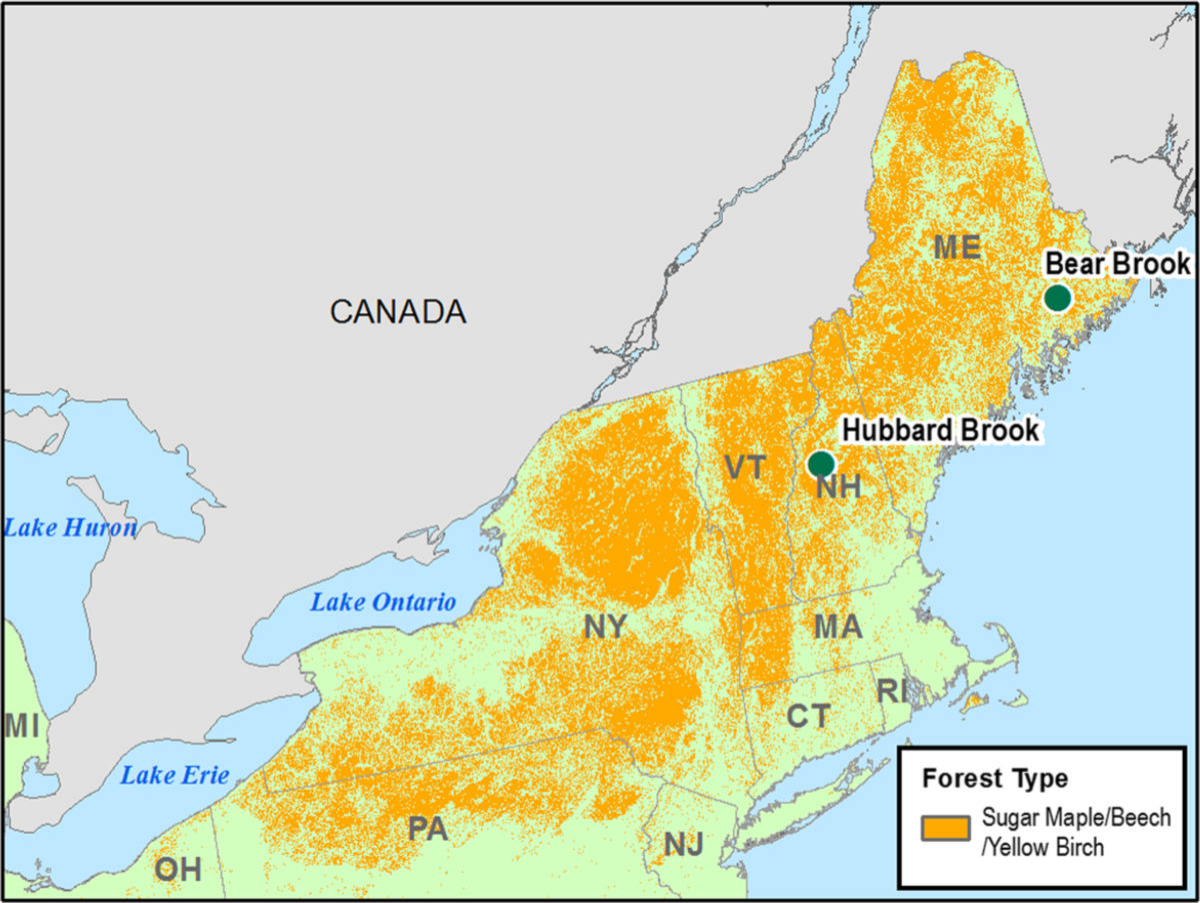
Geographical spread of the sugar maple (*Acer saccharum*), beech (*Fagus grandifolia*), and yellow birch (*Betula alleghaniensis*) (SMBYB) forests in the Northeastern U.S., including the locations of the two sites modeled in this study

The Northeastern U.S. was selected for several reasons: (1) the northeast has historically received some of the highest levels of N and S deposition in North America (NADP 2015), (2) northeastern forests in the USA provide a range of ecosystem services locally and regionally (USFS NSRC, 2014), and (3) high-quality data sources are available to setup and refine the modeling framework. The sugar maple-beech-yellow birch (SMBYB) forest system was selected to represent hardwood forests in this study to test the ForSAFE-Veg model. These forests are a dominant forest type in the Northeastern U.S. (Lovett and Mitchell 2004; Fig. 1), consisting of tree species including sugar maple (*Acer saccharum*), and yellow birch (*Betula alleghaniensis*) which are sensitive to atmospheric deposition and soil conditions (Long et al. 2009; U.S. Forest Service, 2014). Within the SMBYB forest type, two sites were selected that had detailed enough soil and plant community data required for model development—the Hubbard Brook Experimental Forest (HBEF) in New Hampshire and the Bear Brook Watershed in Maine (BBWM).

### 2.2 Description of Sites

#### 2.2.1 Hubbard Brook Experimental Forest

The Hubbard Brook Experimental Forest (HBEF) covers 3120 ha in the southern White Mountain Region in central New Hampshire, USA (43° 56′ N, 71° 45′ W) (Johnson et al. 2000). The climate is humid continental, receiving an average of 1310 mm of precipitation annually, with 25–30% occurring as snow (Lovett et al. 1996). Average January and July temperatures are − 9 °C and 19 °C, respectively, and the growing season spans approximately May 15 to October 1. The Experimental Forest includes several watersheds, all of which were logged in 1920 (Johnson et al. 2000; Likens et al. 1994; Whittacker et al. 1974). HBEF also experienced a hurricane in 1938, during which approximately 20% of the standing biomass was wind-thrown and salvaged (Aber and Driscoll 1997). Current forests are predominantly composed of northern hardwoods, sugar maple, American beech (*Fagus grandifolia*), and yellow birch, with red spruce (*Picea rubens*), balsam fir (*Abies balsamea*) and white birch (*Betula papyrifera*) also abundant at higher elevations and on rock outcrops (Johnson et al. 2000; Whittacker et al. 1974). The soils of HBEF are well-drained heterogeneous Spodosols (Haplorthod), which developed from glacial till from the Wisconsinian glacial period (Johnson et al. 2000). The average depth of the soil to the C horizon is approximately 60 cm (Johnson et al. 1991b). The soils are relatively base-poor, with low effective base saturation (around 9% in the mineral soil) and pH between 4.1 and 4.7. The organic layer is relatively thick (7 cm) and acidic (pH 3.9) yet with 50% base saturation, with a moderate C to N ratio (22.9) (Johnson et al. 1991b).

This current study was conducted using data from a 10 m × 50 m plot in the lower elevation mature hard-wood forest (572 m of altitude) just west of watershed (W6), the biogeochemical control watershed of HBEF (Johnson et al. 2000; Likens et al. 1994). This plot was selected because it was the only location for which both plant community and soil biogeochemistry data were available. This plot and the immediate surrounds are similar to W6 and have been used to characterize throughfall (Lovett et al. 1996; Likens et al. 1998; Likens et al. 1994) and soil solution chemistry (Johnson et al. 2000; Dahlgren and Driscoll 1994; Driscoll et al. 1998) for W6.

#### 2.2.2 Bear Brook Watershed Maine

The BBWM watershed is a long-term paired watershed experiment located in eastern Maine (44° 52′ N, 68° 06′ W), approximately 60 km from the Atlantic Ocean (SanClements et al. 2010; Rustad et al. 1994). It consists of two watersheds, East Bear (EB) and West Bear (WB). EB is 11 ha and serves as the reference watershed (Fernandez et al. 2010). West Bear is 10.3 ha and has been treated bimonthly since November 1989 with 28.8 kg S/ha per year and 25.2 kg N/ha per year as ammonium sulfate ((NH_4_)_2_SO_4_) (Navrátil et al. 2010; Fernandez et al. 2003). Both watersheds are headwater, first-order streams, and are located on the southeastern slope of Lead Mountain, spanning an elevation range of 265–475 m (Rustad et al. 1994; Cosby et al. 1996). The mean annual precipitation at BBWM is 1160 mm (Navrátil et al. 2010), with 25% falling as snow (Cosby et al. 1996). The mean annual temperature is 4.9 °C (Fernandez et al. 2010). Vegetation in EB and WB consists of predominantly second-growth forests that established following the harvesting of the watersheds around 1945 (Norton et al. 1999), although the stand age of BBWM is uneven due to selective harvesting over the past century (Rustad et al. 1994). Forests in the lower portions of the catchments consist predominantly of northern hardwoods (sugar maple, beech, yellow birch, and red maple (*Acer rubrum*) (Rustad et al. 1994). Vegetation at higher elevations and on steeper slopes is mainly dominated by red spruce, balsam fir, and white pine (*Pinus strobus*). Hardwood, softwood, and mixed hardwood-softwood vegetation are found on approximately 34, 25, and 40% of the catchment area, respectively (Elvir et al. 2010). Soils in EB and WB consist mainly of coarse-loamy, mixed, frigid Typic Haplorthods formed from Wisconsinan till that averages 0.9-m thick (range of 0–5 m; Fernandez et al. 2003; Norton et al. 1999). The bedrock in BBWM consists of quartzite and gneiss with granitic intrusions (Norton et al. 1999). The untreated soils within the watershed are acidic and generally base-poor with effective base saturation ranging from 5 to 16.8% and pH values from 3.7 to 4.5, depending on the horizon (SanClements et al. 2010). The forest floor ranges from 2 to 20 cm in depth, with pH between 2.9 and 3.4 and base saturation between 40 and 58% (I. Fernandez, personal communication).

This study focused on the hardwood forests within EB and WB of BBWM. Soils (SanClements et al. 2010; Fatemi et al. 2012) and vegetation (Eckhoff and Wiersma 2002) have been characterized by multiple plots and soil pits within the hardwood forest components of the two watersheds, and data restricted to these locations were used in this study.

### 2.3 Model Description

#### 2.3.1 ForSAFE

The ForSAFE model simulates the cycles of water, C, nutrients, and other chemical elements in a forest ecosystem (Wallman et al. 2005; Belyazid 2006; Zanchi et al. 2014). It includes dynamic descriptions of tree photosynthesis and growth, litter decomposition, tree and soil hydrology, and soil and soil solution chemistry. ForSAFE is modularly constructed. The module for tree photosynthesis, growth, phenology, transpiration, and uptake is based on the PnET model (Aber and Federer 1992). The module for soil and soil solution chemistry is based on the SAFE model (Warfvinge et al. 1993, Alveteg et al. 1998a, 1998b), which contains a module for silicate mineral weathering based on the PROFILE model (Warfvinge and Sverdrup 1992). Additional details on the ForSAFE model can be found in previous publications (esp. Wallman et al. 2005, Belyazid 2006, Belyazid et al. 2006).

#### 2.3.2 The Ground Vegetation Composition Model Veg

The Veg model uses a set of environmental variables to simulate the composition of a plant community at the herbaceous layer (up to a height of 1.8 m) in response to soil moisture, soil solution N and pH, light, temperature, and competition through root depth and shading height (Sverdrup et al. 2007). Details on the Veg model can be found in previous publications (esp. Belyazid 2006, Sverdrup et al. 2007). While keeping the concept and main structure of Veg unchanged, both the parameterization of the plant niches and their numerical descriptions were modified for this study. This was done to harmonize the description of the different niches and to make the units of the niche parameters consistent with the units of the environmental drivers.

The modified version of Veg characterizes each plant species by a set of fundamental niches (Sverdrup et al. 2007). The temperature niche within which a plant can exist is defined by a minimum and a maximum temperature window. Soil N, pH, and moisture and shade tolerance niches are described by an optimum class ranking with tolerance tails. These niches are numerically described using normalized Gaussian distributions (Eq. 1):

(1) $$Resp(driver,opt,var)=e^{-}\frac{{\left(\text{driver-opt}\right)}^{2}}{\text{var}}$$

where *driver* can be soil solution pH, soil solution N (mg/l), soil moisture saturation (given as the fraction of total saturation), or the fraction of above canopy light reaching the forest floor vegetation. *opt* refers to the optimal value of a given driver at which a plant’s specific response curve is maximal, and *var* denotes the tolerance (i.e., the degree to which plant responds to changes in a driver as it deviates from the optimal value) of a plant to variations in the driver (see the “Ground Vegetation Data” section below for a description of how the plant fundamental niches were determined for this study). This modified version of Veg differs from the original version of the model in that all environmental responses are consistently described with Gaussian responses, and each set of niche parameters is given in the units of its respective environmental driver. The suitability of a site for a given species is obtained by multiplying the four specific responses (pH, N, soil moisture, and light). Each plant is given a rooting depth that gives it access to different soil layers, and thereby response to different values of the soil drivers (pH, N, and moisture). The plants also compete for light, with the taller plants shading the shorter ones, thereby gaining an advantage. The suitability of the drivers together with the shading height give each plant a specific strength that is compared with all the other plants to assign a relative surface cover to each.

### 2.4 Model Inputs

ForSAFE-Veg requires data pertaining to soil physical, chemical, and hydrological properties as well as time series of atmospheric deposition and climatic data. The model also uses parameters specific to canopy vegetation, herbaceous layer vegetation, soil minerals stoichiometry, and organic matter decomposition. Appendix 1 and 2 contains a detailed description of model inputs for ForSAFE and Veg, respectively.

#### 2.4.1 Atmospheric Deposition and Climatic Data

The ForSAFE model requires monthly climate and deposition data. For lack of long-term monthly resolution of deposition data over the entire simulation period (1900–2100), the model distributes yearly values evenly over the year. Total deposition (wet plus dry deposition) was estimated by concatenating data for the historical (1900–1993) and contemporary (1994–2009) time periods. Historical total N, S, base cation (calcium (Ca^2+^), magnesium (Mg^2+^), potassium (K^+^), sodium (Na^+^)), and chloride (Cl^−^) deposition at both sites was developed from hindcasted wet deposition for the Northeastern U.S. based on Gbondo-Tugbawa and Driscoll (2003) and the Community Multi-scale Air Quality (CMAQ) model dry to wet ratios (R. Dennis, personal communication). The historical wet deposition estimates were scaled to 3-year average (1994–1996) National Atmospheric Deposition Program (NADP) National Trends Network (NTN) wet deposition at each site (NADP 2015). Historical deposition estimates for BBWM could not account for observed soil solution sulfate (SO_4_^2−^) concentrations. Therefore, BBWM wet S deposition drawn from Fernandez et al. (2003) and wet-dry ratios from Cosby et al. (1996) replaced the total S deposition estimates derived from the hindcasted values. In addition, to account for the experimental additions of N and S in WB (Kahl et al. 1993), 28.8 kg S/ha per year and 25.2 kg N/ha per year (as NH_4_^+^) were added to the annual deposition estimates from 1989 to 2009. The total pre-industrial deposition (average of 1850–1852) for both sites was also estimated using the combination of hindcasted wet deposition and dry to wet ratios scaled to each site. Contemporary total N, S, base cation, and Cl^−^ deposition at both sites were estimated using the 1994–2009 wet deposition data from NADP NTN (NADP 2015) and CMAQ dry to wet ratios (R. Dennis, personal communication).

For climate, total monthly precipitation and monthly average minimum and maximum temperatures from 1900 to 2009 were derived from the PRISM historic (PRISM 2013a) and recent years (PRISM 2013b) climate datasets. Annual atmospheric CO_2_ concentrations were from the Representative Concentration Pathways (RCP) database version 2.0.5 for 1900 to 2009 (RCP Database 2013).

#### 2.4.2 Soil Data

The soil at HBEF was represented by eight layers (O, A, E, Bhs, Bs1, Bs2, Bs3, and Cd), which were described from soil column samples collected in 1997. Soil texture was estimated by laser diffraction with Partica LA 950 (J. Aherne, personal communication) and combined with literature data (Federer 1992; Balland et al. 2008; Phelan et al. 2014; Warfvinge and Sverdrup 1992; Johnson et al. 1991a, 1991b; Yanai et al. 2006) to provide the numerical inputs used by the model; these are given in Tables 2 and 3 in Appendix 1.

Soil samples for EB were collected in 2010 from 3 hardwood forest locations and served, together with data from SanClements et al. (2010), as the basis to describe the soil with five layers (O, B1, B2, B3, and C). The soil at WB was assumed to be generally similar to EB, with differences in texture, cation exchange capacity (CEC), and base saturation (BS). A detailed description of the soil parameters is given in Tables 4, 5, and 6 in Appendix 1.

#### 2.4.3 Tree Data

Parametric data for photosynthesis, evapotranspiration, allocation, and phenology were derived from earlier studies of northern hardwood forests (Aber and Driscoll 1997). Tree species composition was used to parameterize tissue nutrient requirements using weighted averages according to the proportion of the respective trees (Tables 7 and 8, Appendix 1). At HBEF, the forest biomass was composed of 32% sugar maple, 29% American beech, and 39% yellow birch (2007 values). At EB and WB, the forest was dominated by American beech, red spruce, sugar maple, and yellow birch, with occurrences of other tree species (Eckhoff and Wiersma 2002).

#### 2.4.4 Ground Vegetation Data

Parameterization of the plant species’ physiological traits and environmental niches was carried out during a two-day Expert Plant Ecologist Workshop involving the authors, seven of whom are professional botanists familiar with the local flora. Parameterization involves, first, identifying which species are to be included in the simulation and, second, assigning niche values (i.e., central tendency and distribution tails) along six niche axes presented above. Understory plant species were identified based on presence in the hardwood forest component of EB or WB, the HBEF plot, or in the understory of U.S. Forest Service Forest Inventory and Analysis (FIA) SMBYB forest plots (restricted to plants that were present in at least 5% of plots and/or representing greater than 5% of plant cover in a single plot). Twenty-two additional SMBYB plant species were added to the list by consensus of the plant ecologists. These species were identified based on their occurrence in unique edaphic conditions and/or cultural significance. The final list of SMBYB understory plant species considered for this study consisted of 181 species. During the workshop, each species on this list was reviewed and parameterized by the ecologists, with the parametrization focusing on the six separate niches axes. The physiological and environmental niche parameters were categorized by classes (see Table 9 in Appendix 2 for a key to how the classes relate to actual field parameters). The resulting plant species parameter table was then trimmed down to the 45 plants present at the HBEF plot or the hardwood forests of BBWM, and these 45 species were used for the uncalibrated (also referred to here as blind) simulations and calibration of the vegetation traits and niches. The final Veg plant species parameter table is presented in Table 10 in Appendix 2.

### 2.5 Model Calibration Procedure

Model calibration was conducted in two steps, calibration of the biogeochemical part of the model (ForSAFE) followed by calibration of the vegetation response model (Veg).

The calibration of ForSAFE consisted of back-calculating the historical levels of exchangeable base cations and soil organic matter (including C and N) required for the model to simulate currently observed values of base saturation and soil organic matter (Belyazid 2006). An iterative dynamic routine is used to set the level of base saturation, soil organic C, and soil total N at the year 1900 (the start year for the simulations) so that simulated values of these variables matched their respective current field observations. No additional parameters were calibrated in ForSAFE.

Calibration of Veg involved the adjustment of the niche values by modifying the optima and/or variance to ensure the projected cover more closely resembled observed cover on a species-by-species basis (Appendix 2). The calibration of Veg involved a three-step process applied to plant species with a more than 5% error in predicted cover. First, drivers that caused a sub-dominant plant (i.e., a plant that occupied less than 10% cover) to be modeled as dominant (i.e., cover > 20%) were identified, reviewed in the literature, and adjusted within the literature constraints to reproduce the observed plant cover. Secondly, drivers that suppressed the simulated cover of a measured dominant plant were identified, reviewed in the literature, and modified to reproduce the observed cover. Lastly, the same procedure was repeated consecutively for sub-dominant (i.e., plants that covered 10–20% of the area). Out of the 45 plants modeled, 12 light classes were revised, 7 N classes, 4 pH classes, 2 shading heights, and 1 moisture class were revised. The average modifications made to the expert defined classes of optimal values were 1.2, 0.6, 0.6, 1.3, and 0.5 classes for light, N, pH, shading height, and moisture, respectively. The calibrated parameter classes are given in Table 9, Appendix 2.

### 2.6 Model Performance Evaluation Metrics

A set of statistical metrics was used to evaluate the performance of the biogeochemical components of the model (ForSAFE) when compared with observed field data.

To determine whether the model over- or underestimated the observed biochemical indicators, three metrics were used: (1) the normalized average error (NAE, Eq. 2), which gives an estimate of bias in the mean and requires the least data, is suitable for comparing the limited records of tree biomass (*n* ≤ 20); and (2) the slope of the relationship between predicted (*y*-axis) and observed (*x*-axis) values with a zero intercept using annual averages (i.e., the 1:1 line, a slope value inferior to 1 indicates an underestimation by the model), which allows for bias to change along the range of a given value, but requires more data. The slope of the 1:1 line was used for the soil solution chemical indicators and was complemented with the standard error (SE) and the correlation factor (Cr) to quantify the spread in the scatter. This method is preferred when sufficient data points were available (i.e., *n* > 20).

(2) $$NAE=\frac{\left(\overset{‾}{P}-\overset{‾}{O}\right)}{\overset{‾}{O}}$$

where $\overset{‾}{P}$ and $\overset{‾}{O}$ are the means of the predicted and measured values (Janssen 1995).

A third measure, the normalized root mean square error (NRMSE), was used to estimate the total error in the simulations when data were sufficient (*n* > 20, Eq. 3), and could be used to relate the temporal changes of the observed and modeled data:

(3) $$\mathrm{NRMSE}=\frac{1}{\overset{‾}{O}}\cdot \sqrt{\frac{\sum_{i=1}^{N}\;{\left(P_{i}-O_{i}\right)}^{2}}{N}}$$

where $\overset{‾}{O}$ is the mean of the observed values, and N is the number of observed/modeled pairs O and P (Janssen 1995). The NRMSE is a strict difference metric, as it amplifies the larger differences within individual observed/modeled pairs, and thus gives a less aggregated value than other metrics (for example the NAE which compares the means of the populations rather than the individual observed/modeled pairs).

The metric used for evaluating the performance of the ground vegetation composition model Veg was the Czekanowski similarity index (CzI), given by Eq. 4 (Bray and Curtis 1957). CzI, also called the Sörensen index or the reverse Bray-Curtis index, was selected because it is one of the more reliable indices of similarity as it accurately integrates the inter-community overlaps in a symmetrical system (such as the normalized covers used here) and does not penalize nor overly account for the non-dominat species (Bloom (1981), Wolda (1981), Boesch (1977)). CzI = 1 indicates a perfect fit, and the smaller the CzI, the lesser the similarity between the modeled and the observed plant communities:

(4) $$CzI=1-\frac{\sum |P-O|}{\sum (P+O)}$$

## 3 Results

### 3.1 Simulating Soil Solution Chemistry

The simulated soil solution concentrations of Cl^−^, Na^+^, SO_4_^2−^, inorganic N, and base cations (Bc^2+^ = Ca^2+^+ Mg^2+^+K^+^), as well as soil solution pH, are compared with corresponding measurements both as time series plots and corresponding statistical metrics (Figs. 2, 3, and 4 for HBEF; Figs. 5, 6, and 7 for EB; and Figs. 8, 9, and 10 for WB).

Model performance, evaluated through the agreement between the simulated and measured soil solution chemical indicators, varied considerably between indicators and depths among the three sites. A general trend is that the model performed better when evaluated through the annual averages using the slopes of the 1:1 lines and the corresponding standard errors and correlation coefficients. For the biggest majority of tested indicators, the slope of the 1:1 line was within 0.7 to 1.3 (± 0.3 from the optimal value of 1). The standards errors were generally small, while the correlation coefficients ranged from strongly positive to weak and even negative in exceptions. The standard deviation from the annual averages was comparably large between the measured and the modeled values.

Evaluated through the NRMSE (i.e., taking into account the highest temporal resolution of comparable data), the model performed moderately well but less so than with the previous indicator. There was no more expressed difference between the sites, given the large difference in observed data density, than within the sites among the simulated soil horizon. The modeled data showed more variability than the observations. The model was, however, able to reproduce the observed ranges, and in many cases, also the observed trends and oscillations.

**Fig. 2:**
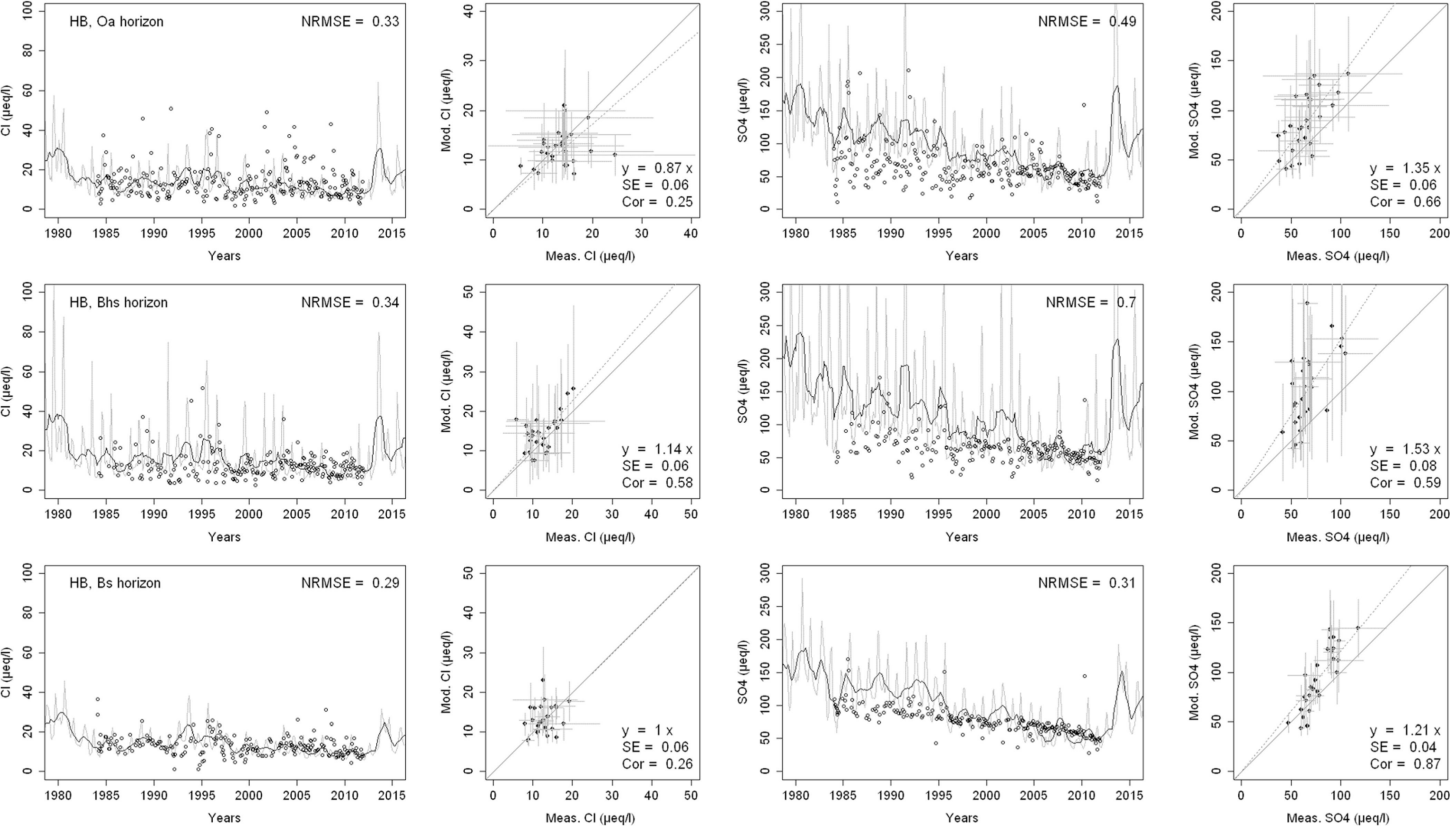
Modeled and measured soil solution concentrations of chloride (Cl^−^) and sulfate (SO_4_^2−^) at three depths at Hubbard Brook Experimental Forest (HBEF). In the first and third columns, the dark lines show modeled 12-month moving averages, the gray lines show modeled monthly values, and the points are field measurements. The second and fourth columns show 1:1 correlations of yearly medians and standard deviations of modeled and field measured concentrations

The concentrations of Cl^−^ in the soil solution were well reproduced by the model at the three sites and all depths (Figs. 2, 5, and 8). The 1:1 slopes were within ± 0.3 at all sites and depths, except for the 60-cm depth at WB. The standard errors were smaller at HBEF (= 0.06) but higher at EB and WB, where data density was markedly lower. The coefficients of correlation were above 0.3 at all sites and depths, but the discrepancies were due to different reasons at the different sites. The model produced higher seasonal variation at EB and WB, while it underestimated it at HBEF. This is reflected in the NRMSE values that lie around 0.3 with three exceptions (25-cm and 60-cm depth at EB, and 60-cm depth at WB). Despite the large seasonal variation of both the modeled and measured data, the NRMSE values showed a relatively good agreement at the higher time resolution. The highest recorded NRMSE occurred at the depths with the lowest number of measurements (60 cm at EB and WB).

Soil solution concentrations of SO_4_^2−^ showed a distinct decline over the measurement period due to changes in an atmospheric deposition at HBEF and EB (Figs. 2 and 5). At WB, on the other hand, the experimental additions of (NH_4_)_2_SO_4_ produced a clear increase in SO_4_^2−^ concentrations (Fig. 8). The model captured the SO_4_^2−^ patterns and ranges moderately well at all sites, particularly when considering the 1:1 comparison and associated standard errors. The correlations between modeled and observed [SO_4_^2−^] were clearly stronger than for Cl^−^, except for the 60-cm depth at EB, showing that the year-to-year changes were well captured by the model. However, the model exaggerated the seasonal variations at HBEF, as shown by the higher NRMSE values.

**Fig. 3:**
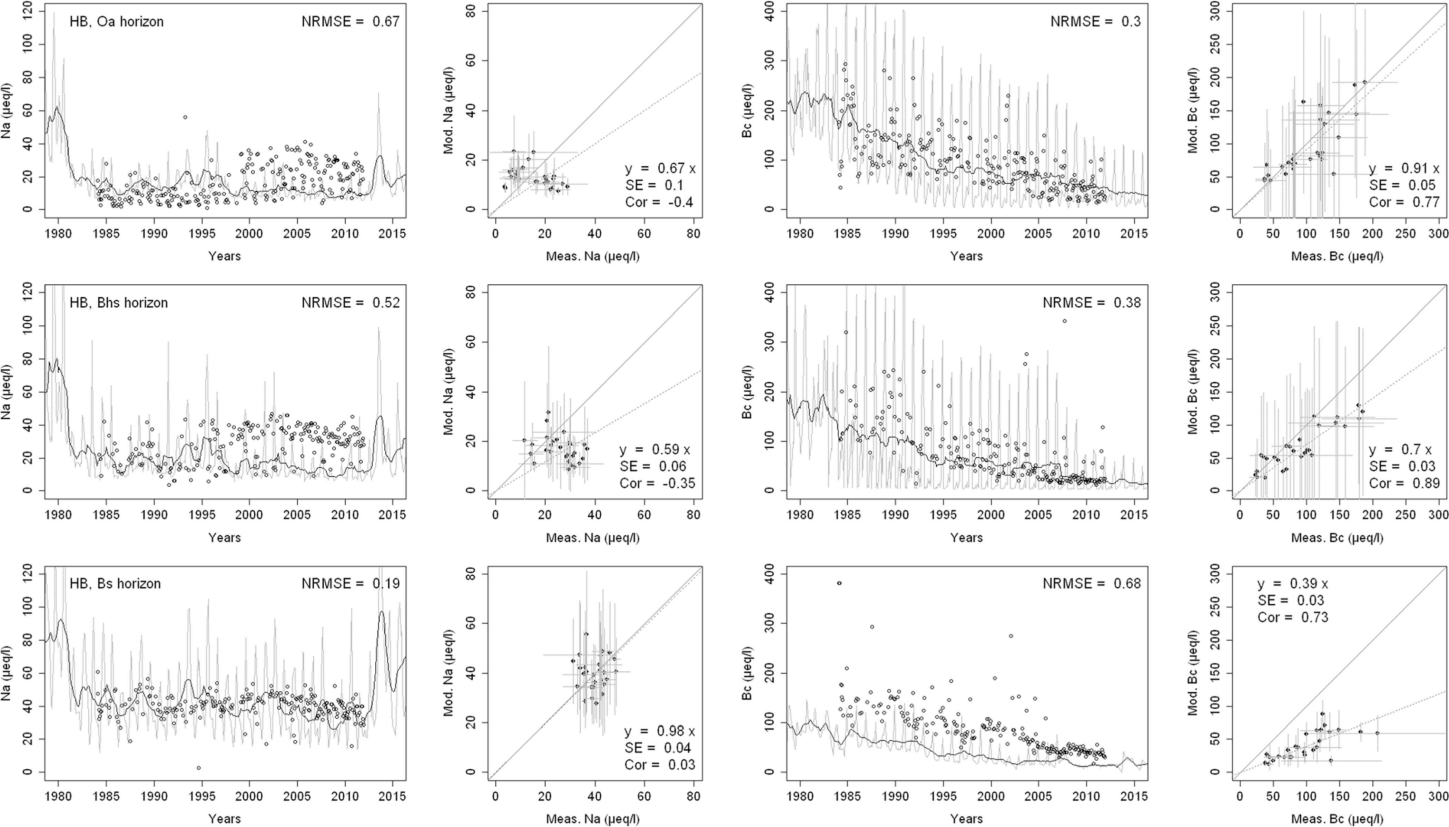
Modeled and measured soil solution concentrations of sodium (Na^+^) and base cations (Bc^2+^; Ca^2+^, Mg^2+^ + K^+^) at three depths at Hubbard Brook Experimental Forest (HBEF). In the first and third columns, the dark lines show modeled 12-month moving averages, the gray lines show modeled monthly values, and the points are field measurements. The second and fourth columns show 1:1 correlations of yearly medians and standard deviations of modeled and field measured concentrations

The simulated concentrations of Na^+^ (Figs. 3, 6, and 9) compared well with both the monthly and yearly measurements. While the agreement between observed and modeled Na^+^ improved with depth at HBEF, it showed the opposite pattern at EB and WB. At HBEF, the measured Na^+^ in the Oa and Bhs horizons showed a step increase from around the year 2000 and onwards. This increase was not captured by the model, causing an underestimation of the simulated concentrations. In the Bs horizon at HBEF, the model agreed well with the measurements (Fig. 3). At EB and WB, the simulated concentrations of Na+ agree well with the observations at both 5 cm and 25 cm depths where the measured data are more dense (Figs. 6 and 9). At 50-cm depth, where the measured data is sparse, the model overestimated Na^+^.

The ranges of base cations (Bc; Ca^2+^, Mg^2+^, and K^+^) concentrations in the soil solution, indicated by the 1:1 slopes, were well simulated by the model (Figs. 3, 6 and 9), with two exceptions: in the Bs horizon at HBEF (Fig. 3) and at the 25-cm depth at WB (Fig. 9). The model predicted a faster increase in Bc following the addition of (NH_4_)_2_SO_4_ at WB (Fig. 9). Looking at the higher time resolution, the model captures the declining trend in Bc at HBEF (Fig. 3) and at the shallower depth at EB (Fig. 6). The simulated concentrations of Bc increased consistently with depth, with the exception of the BS horizon at HBEF. As seen with the other elements above, the exaggerated seasonal variation by the model reduced its performance when evaluated by the NRMSE. The correlations of the annual medians of Bc varied greatly, from strongly positive at HBEF to weak in the deeper soil layers in WB and even negative at EB.

**Fig. 4:**
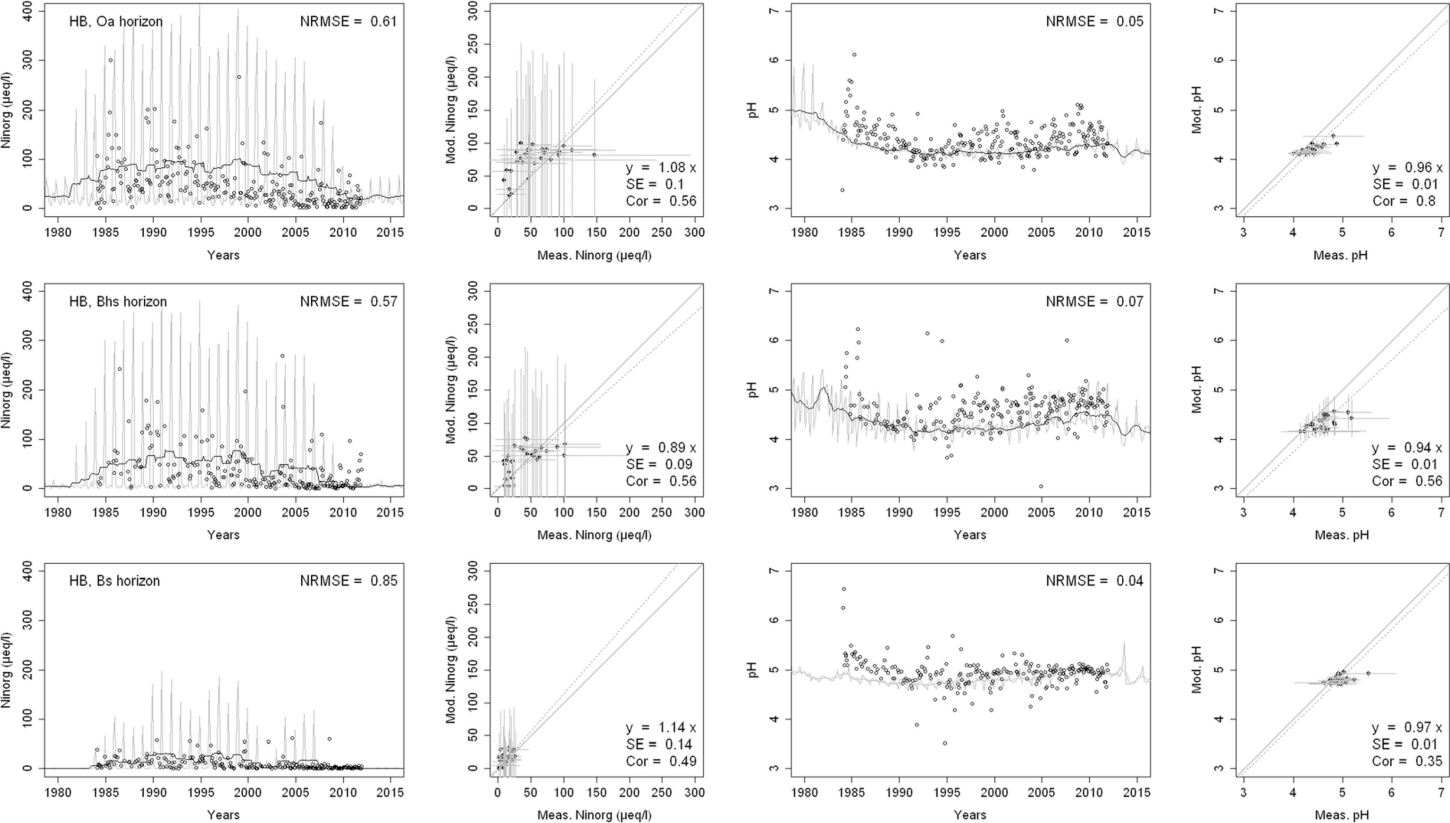
Modeled and measured soil solution concentrations of inorganic nitrogen (NO_3_^−^ + NH_4_^+^) and pH at three depths at Hubbard Brook Experimental Forest (HBEF). In the first and third columns, the dark lines show modeled 12-month moving averages, the gray lines show modeled monthly values, and the points are field measurements. The second and fourth columns show 1:1 correlations of yearly medians and standard deviations of modeled and field measured concentrations

**Fig. 5:**
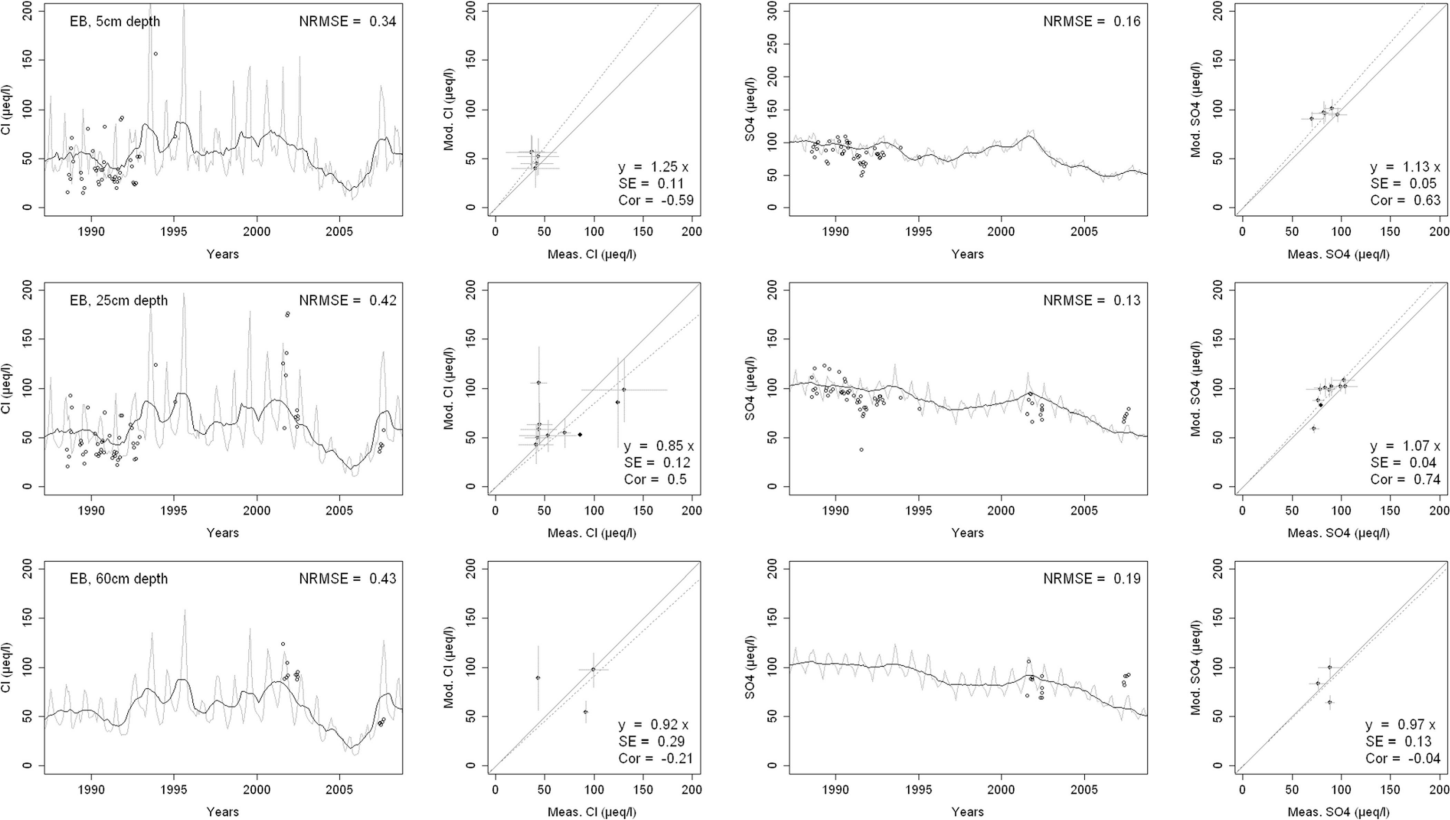
Modeled and measured soil solution concentrations of chloride (Cl^−^) and sulfate (SO_4_^2−^) at three depths at East Bear (EB) watershed. In the first and third columns, the dark lines show modeled 12-month moving averages, the gray lines show modeled monthly values, and the points are field measurements. The second and fourth columns show 1:1 correlations of yearly medians and standard deviations of modeled and field measured concentrations

**Fig. 6:**
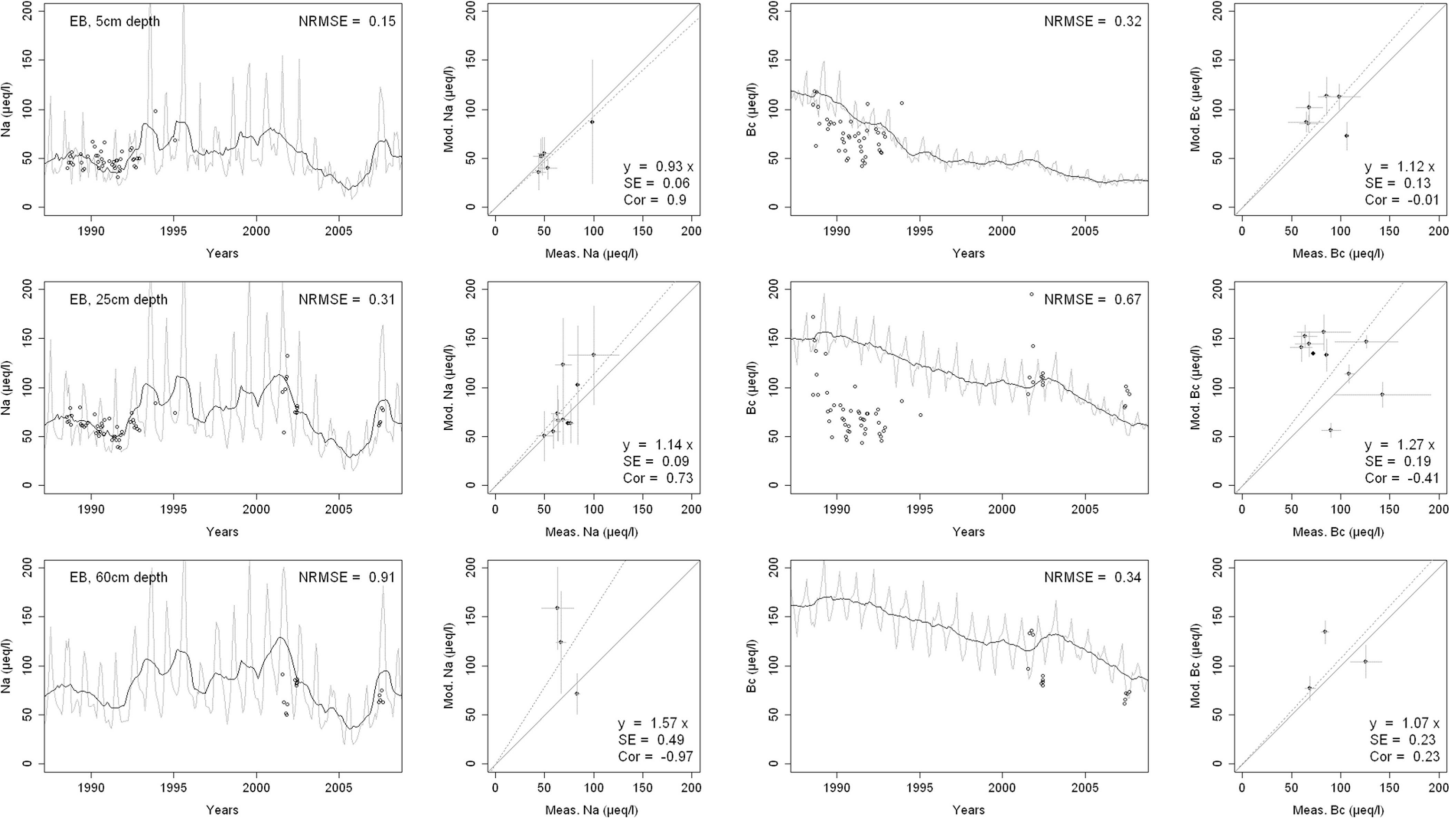
Measured and modeled soil solution concentrations of sodium (Na^+^) and base cations (Bc^2+^; Ca^2+^, Mg^2+^,+ K^+^) at three depths at the East Bear (EB) watershed. In the first and third columns, the dark lines show modeled 12-month moving averages, the gray lines show modeled monthly values, and the points are field measurements. The second and fourth columns show 1:1 correlations of yearly medians and standard deviations of modeled and field measured concentrations

**Fig. 7:**
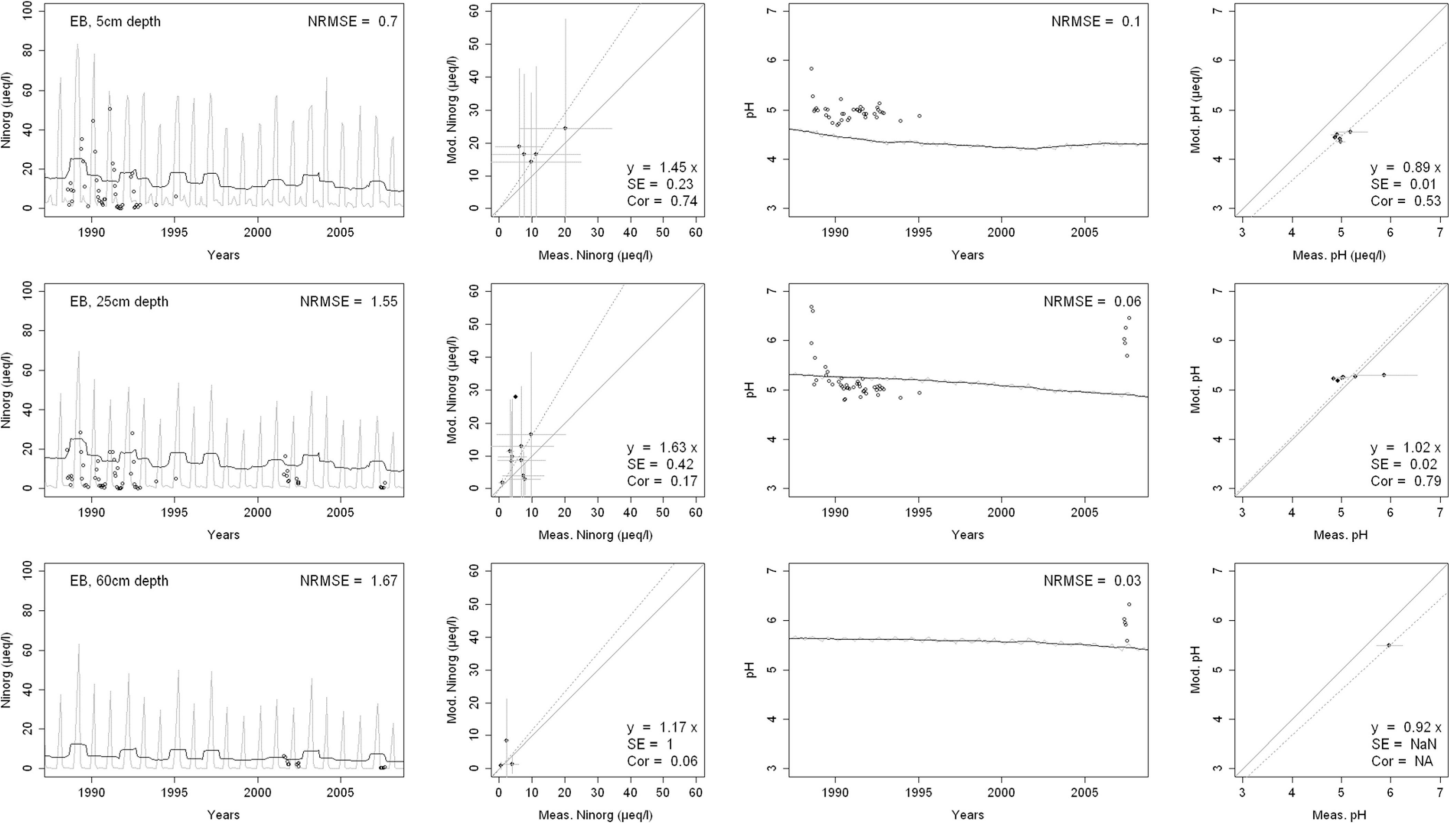
Measured and modeled soil solution concentrations of inorganic nitrogen (NO_3_^−^ + NH_4_^+^) and pH at three depths at East Bear (EB) watershed. In the first and third columns, the dark lines show modeled 12-month moving averages, the gray lines show modeled monthly values, and the points are field measurements. The second and fourth columns show 1:1 correlations of yearly medians and standard deviations of modeled and field measured concentrations

**Fig. 8:**
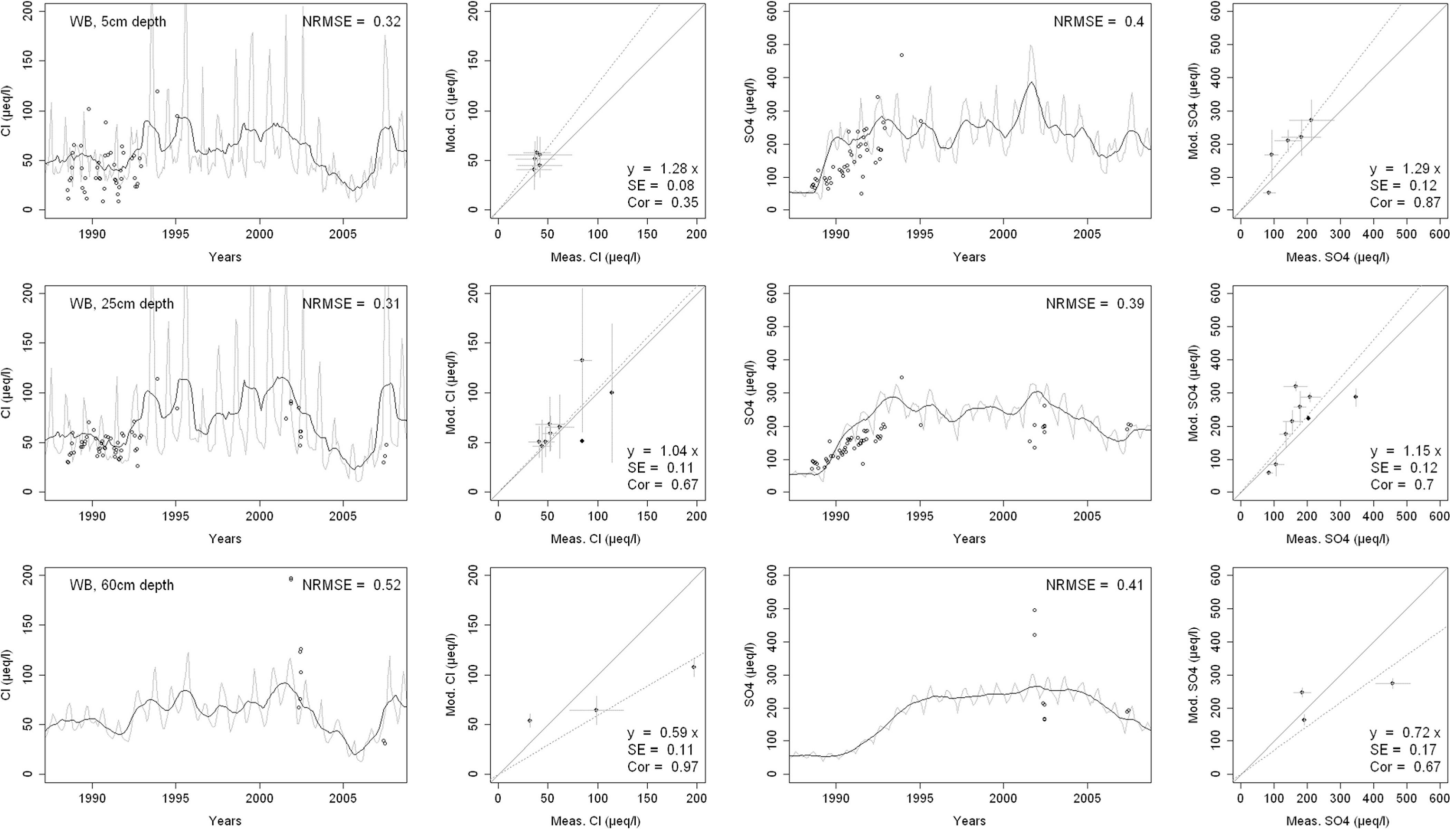
Modeled and measured soil solution concentrations of chloride (Cl^−^) and sulfate (SO_4_^2−^) at three depths at West Bear (WB) watershed. In the first and third columns, the dark lines show modeled 12-month moving averages, the gray lines show modeled monthly values, and the points are field measurements. The second and fourth columns show 1:1 correlations of yearly medians and standard deviations of modeled and field measured concentrations

**Fig. 9:**
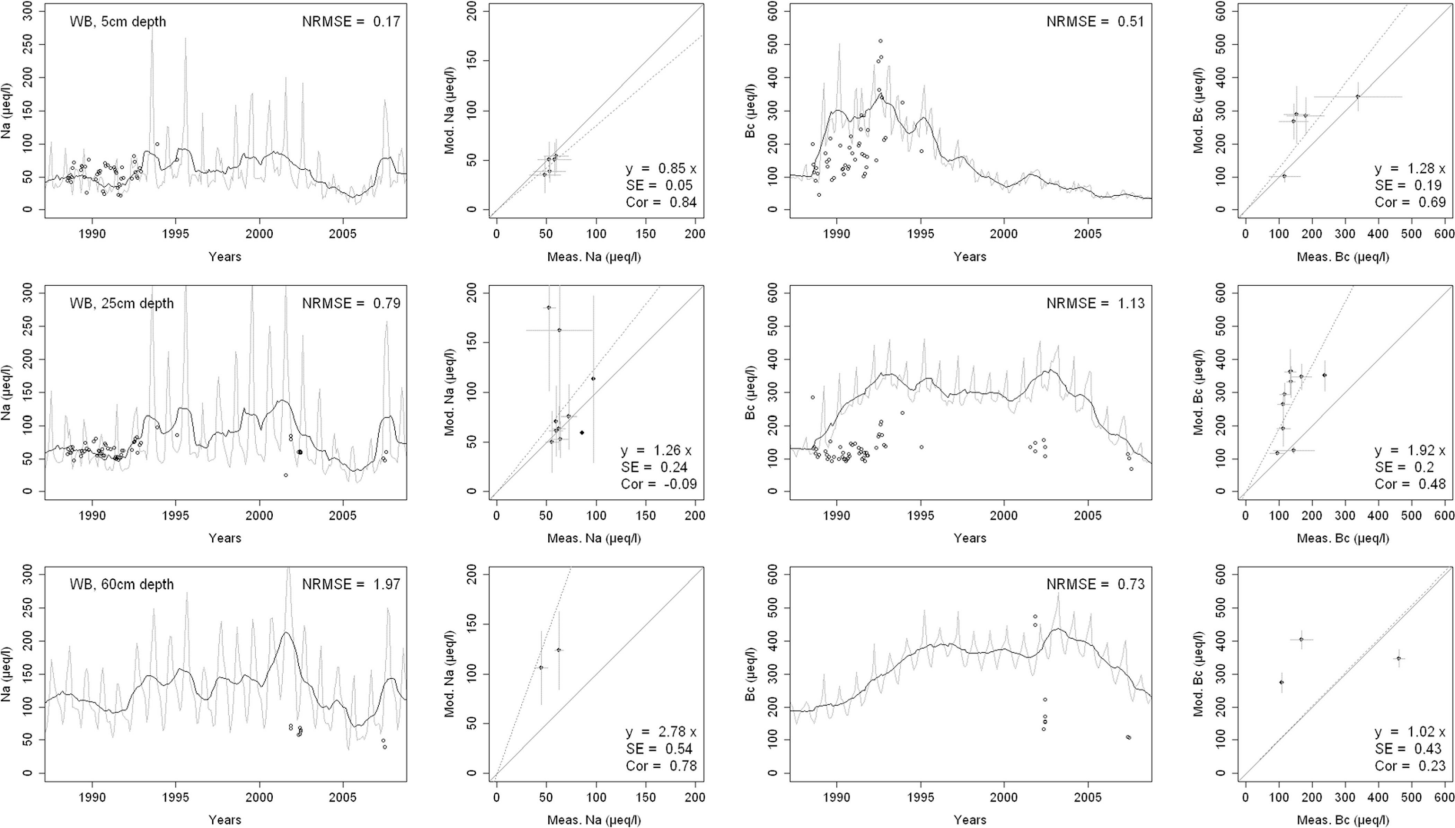
Modeled and measured soil solution concentrations of sodium (Na^+^) and base cations (Bc^2+^; Ca^2+^, Mg^2+^,+ K^+^) at three depths at West Bear (WB) watershed. In the first and third columns, the dark lines show modeled 12-month moving averages, the gray lines show modeled monthly values, and the points are field measurements. The second and fourth columns show 1:1 correlations of yearly medians and standard deviations of modeled and field measured concentrations

**Fig. 10:**
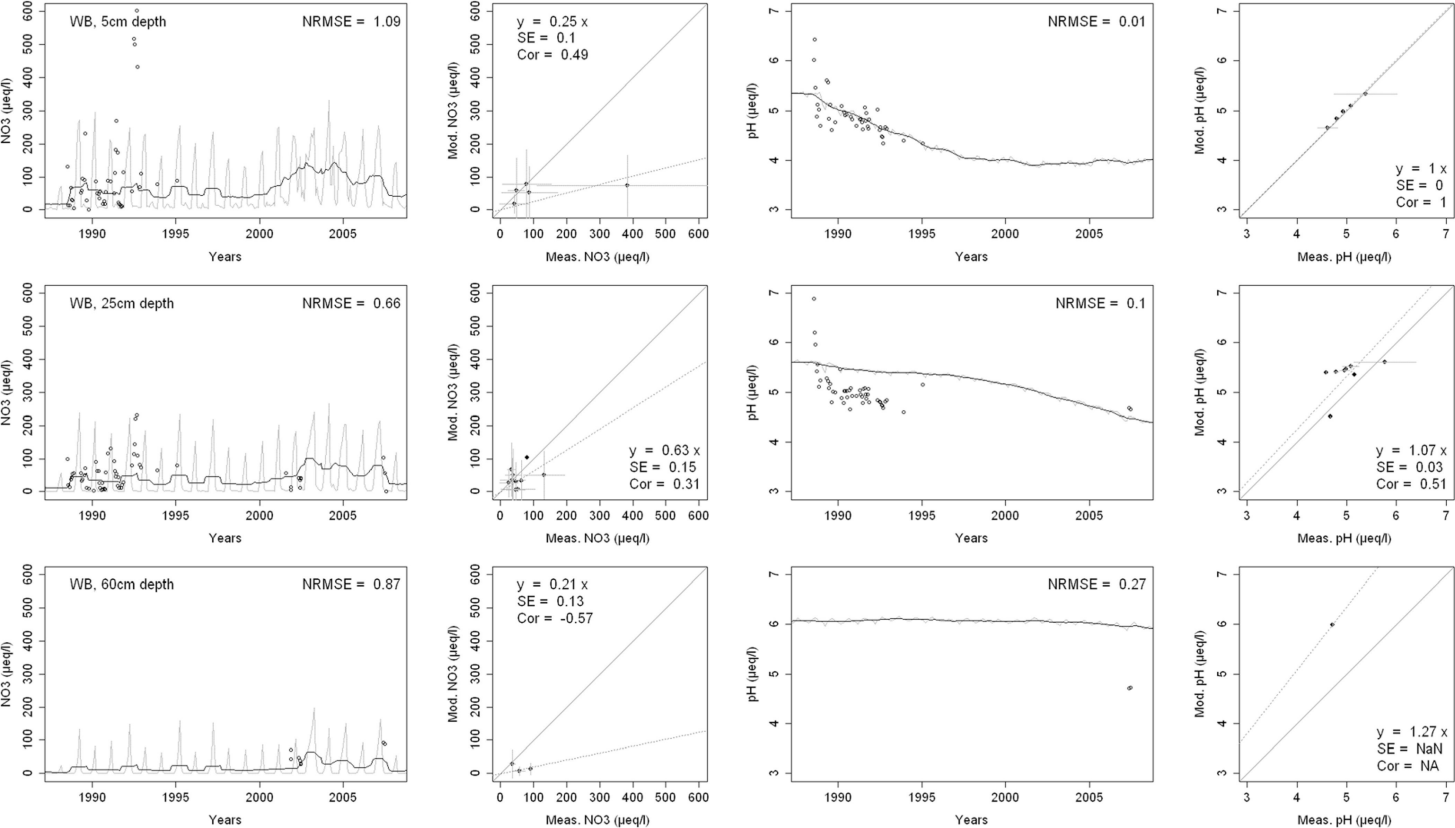
Modeled and measured soil solution concentrations of inorganic nitrogen (NO_3_^−^ + NH_4_^+^) and pH at three depths at West Bear (WB) watershed. In the first and third columns, the dark lines show modeled 12-month moving averages, the gray lines show modeled monthly values, and the points are field measurements. The second and fourth columns show 1:1 correlations of yearly medians and standard deviations of modeled and field measured concentrations

The yearly median concentrations of inorganic N were reasonably well simulated (Figs. 4, 7, and 10). The slopes of the 1:1 curves showed a good agreement with low standard errors at HBEF (Fig. 4), while they indicated an overestimation at EB (Fig. 7) and underestimation at WB (Fig. 10). The correlations between the observed and simulated N were stronger in the shallower soil layers at the three sites. The NRMSE values varied with depth with no clear pattern but indicated a low performance at the higher time resolution. The model produced wider monthly variations than the observations at HBEF (Fig. 4), but these patterns were less obvious at EB and WB. The model captured the decline in N at HBEF, while no trends were apparent at EB. At WB, the experimental addition drove a step increase in concentrations that was more distinct at the 5-cm depth.

The simulated element concentrations and pH values were well within the observed ranges at all sites (Figs. 4, 7, and 10), with very satisfactory 1:1 comparisons and generally low standard errors and low NRMSE. Unlike the individual concentrations, the simulated soil solution pH showed small monthly variations. The model also captured the temporal variation in pH, particularly at HBEF with an initial decline and a later recovery, as well as the observed initial decline at WB (Figs. 4 and 10. The model produced a lesser decline than observed at EB (Fig. 7).

### 3.2 Simulating the Biomass of the Tree Cover

**Fig. 11:**
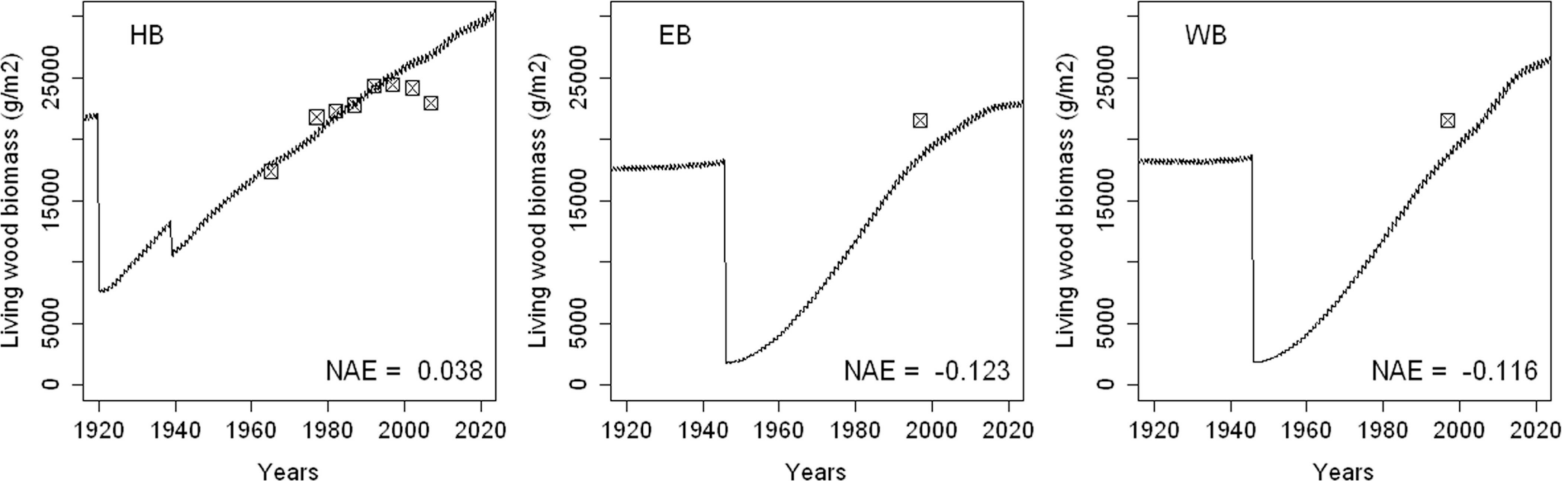
Modeled (line) and measured (squares) standing wood biomass at the Hubbard Brook Experimental Forest (HBEF) and East Bear (EB) and West Bear (WB) watershed sites. The sharp declines show the effects of harvests. The normalized average error (NAE) compares the measured values with their modeled counterparts at the specific measurement years

The model simulated the sizes of tree biomass reasonably well at all three sites with some exceptions (NAE range from + 3.8 to − 11.7%). The model underestimated tree biomass at both EB and WB, and although it simulated tree biomass at HBEF well from 1965 to 1990, it failed to capture the recent decline (Fig. 11). The rates of biomass increment were highest at EB and WB after the clear-cuts of 1945, as seen in the steeper slopes. While biomass leveled off at EB by the end of the simulation period, the growth was simulated to continue increasing at WB after the experimental addition of NH_4_(SO_4_)_2_ since 1989. It was difficult to further judge the performance of the model given the sparseness of the tree biomass data, especially for WB and EB, but model predictions appeared to fit the measurements reasonably well with the above caveats.

### 3.3 Simulating the Composition of the Ground Vegetation Community

**Fig. 12:**
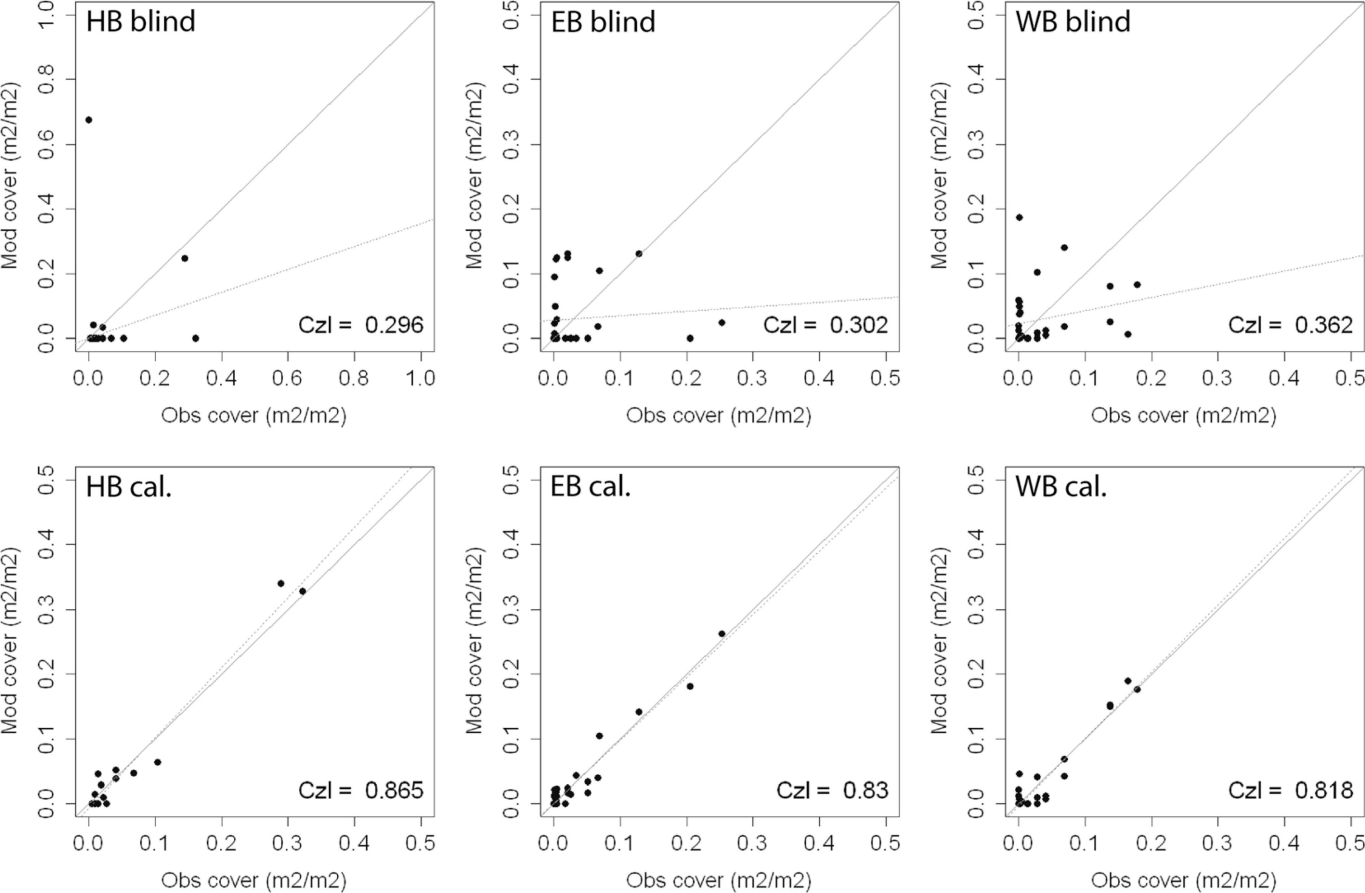
Comparisons of observed relative plant abundance by area cover versus blind (upper row) and calibrated (lower row) model simulations at the Hubbard Brook Experimental Forest (HBEF) and East Bear (EB) and West Bear (WB) watershed sites

The performance of the model in reproducing the observed composition of the understory plant communities was poor before the calibration (blind run in Fig. 12, upper row, Table 1). The community CzI values from the blind simulations were 0.296, 0.302, and 0.362 at HBEF, EB, and WB, respectively, meaning the model correctly predicted roughly a third of the total cover. Calibrating the niches produced a marked improvement in the predictive capacity of the model, with CzI values of 0.865, 0.83, and 0.818 at HBEF, EB, and WB, respectively, and all species at the individual level being within 5% of the observed abundance (Fig. 12, lower row, Table 1). Rare species with a cover less than 5% made up 9 out of 19 plants reported at HBEF, 11 out of 33 at EB, and 8 of 33 at WB (19 species out of the total of the 45 plants modeled). Eleven of these 19 marginal species were reproduced within 5% error under the blind simulations.

The plant community at the plot in HBEF is dominated by two plants (*Viburnum lantanoides* and *Dryopteris intermedia*), occupying 61% of the total area. The blind simulation reproduced the presence of *Viburnum lantanoides* accurately but missed the presence of *Dryopteris intermedia.* In addition, it grossly overestimated the presence of *Dennstaedtia punctilobula*, with this error in prediction being due to the species’ response to the intensity of light at the forest floor. At EB and WB, 59% of the area was shared among three plants (*Betula alleghaniensis*, *Dryopteris campyloptera*, and *Fagus grandifolia*), the last two of which were missed by the blind simulation due to their light response. At WB, the co-dominance was shared among four plants (*Acer pensylvanicum*, *Acer saccharum*, *Dryopteris campyloptera*, and *Fagus grandifolia*), together covering 62% of the area.

## 4 Discussion

### 4.1 Soil Solution Chemistry

The results provide confidence in the model’s ability to simulate soil solution chemistry in response to environmental factors at the three study sites. With no calibration of soil processes other than the back-calculation of the historical base saturation, the overall levels of analytes concentrations and pH in the soil solution were well within the ranges of the observations. While the modeled seasonal variations were wider than observed, the model performance on an annual basis was satisfactory for most analytes and depths. Moreover, the performance of the model was consistent throughout the three sites with different geochemical and deposition conditions. These results support the prospect of expanding the model application to additional sites, particularly with focus on inter-annual rather than intra-annual dynamics.

Consistently through the soil profiles and for all the analytes examined, the model performed better on an annual average basis (as assessed by 1:1 comparisons) than it did on an intra-annual basis (as assessed by NRMSE). The reasons behind this may be found in the temporal, spatial, and structural differences between the model and the empirical data.

Temporally, while the model produces monthly values, which are clearly differentiated in time, field-collected lysimeter data are less obviously associated with a fixed time frame (i.e., a soil solution sample can represent a weekly or a bi-weekly average, depending on the frequency of sampling). Moreover, the resolution of the input data may carry further ambiguity, as is suggested by Cl^−^. Chloride, an arguably conservative ion (Svensson et al. 2012), is an appropriate indicator of Cl^−^ deposition. Yet, because the model uses annual deposition fluxes, it does not capture the pronounced intra-annual variability of Cl^−^ deposition (Lovett et al. 2005; Rustad et al. 1994), thereby weakening the predictive accuracy of soil solution Cl^−^ on a seasonal scale. The same is true for other elements.

Spatially, although the model simulates different soil layers, it assumes a hypothetical uniformity within each soil layer. The simulated soil solution at a given soil layer is represented by an average for the entire layer as defined by the user (8 layers in the case of HBEF and 5 layers in BBWM). Lysimeter samples of the soil solution, on the other hand, are taken at a specific depth or multiple depths and can be strongly influenced by concentration gradients in the soil solution within a soil layer (Vetterlein and Jahn 2004; Schöttelndreier and Falkengren-Grerup 1999). Thus, direct linkages between modeled output and field data remain difficult.

Structurally, the model is built on strict conservation of mass balances for all elements and, for nutrients, in particular, a balance between assimilation by the trees and release through litterfall and mineralization. The cycles of nutrient uptake and litterfall create an exaggerated seasonal variation for nutrient elements. The results of this study clearly show that while the annual mass balance calculations are reliable as indicated by the 1:1 comparisons, there is a clear need for improving the seasonality of physiological processes such as uptake (e.g., Mellander et al. 2006).

Finally, two elements stand out from the discussion above, Na^+^ for its depth patterns and SO_4_^2−^ for its temporal trends. Simulated Na^+^ concentrations improved with depth at HBEF, while they strongly worsened with depth at both EB and WB. Sodium concentrations reflect the combined effects of deposition, hydrology, and weathering, and are mainly dominated by Na^+^ deposition at the shallower depths, with increasing contribution from weathering deeper down in the soil (Na^+^ concentrations are 2.5 times higher in the Bs than in the Oa horizon at HBEF, while 1.5 times higher at EB and only 1.1 times higher at WB). It was noted that the model performed better where Na^+^ concentrations increased strongly with depth (i.e., where Na^+^ resulting from weathering exceeded Na^+^ deposition). Such cases were likely able to dampen any potential errors associated with seasonal uncertainties in Na^+^ deposition.

The rate of decline of SO_4_^2−^ concentrations at HBEF was exaggerated, due to the fact that the SO_4_^2−^ adsorption module within ForSAFE (Martinson et al. 2003) was not activated for the lack of parameter values. The simulated and observed values of SO_4_^2−^ do, however, converge as deposition stabilizes and SO_4_^2−^ in the soil solution leaves the transient stage. The discrepancy in SO_4_^2−^ concentrations is reflected in a delay in the recovery of soil solution pH, apparent in the Oa and Bhs horizons at HBEF.

The disparity in the performance of the model when gauged on an annual versus sub-annual basis implies, notwithstanding the caveats above, that the model is better suited to project annual than sub-annual dynamics. Some dynamics driven by N deposition are known to occur over annual to multi-year time scales, such as species losses from low-level N inputs, which have been shown to occur over decadal time periods (Clark and Tilman 2008). Other dynamics occur over much shorter time periods, such as changes in soil solution nitrate and timing of leaf-out, among others. For these sub-annual responses, we have less confidence in the skill of the model.

### 4.2 Tree Biomass

The model was able to reconstruct the present stand sizes from generic parameters and from the known histories of the stands. Based on the concept by Aber and Federer (1992), the model uses foliar N concentrations to drive photosynthetic rates by capturing light and water use and, specific to ForSAFE, soil base cation and Al concentrations as potentially limiting factors. The model was also able to capture a considerable difference in biomass between WB and EB as a result of the experimental increase in N availability, primarily as a result of foliar N enrichment, in line with the explanation given by Elvir et al. (2010). However, the model underestimated tree biomass at both EB and WB due to the use of the non-calibrated generic parameters. Since the aim of the modeling was to evaluate model applicability on SMBYB stands in the Northeastern U.S., the generic parameters for tree growth and allocation were not modified.

At HBEF, the model failed to reproduce the tree biomass decline by the end of the 1990s (Siccama et al. 2007). Different studies have suggested that a deficiency in Ca^2+^ may be behind the observed decline of northern hardwoods (Hugget et al., 2007; Park et al. 2008; Bailey et al. 2004, Juice et al. 2006), either directly or indirectly through enhancing tree vulnerability to pathogens. Because the reasons behind the decline remain unclear (Siccama et al. 2007), it is difficult to pinpoint specific model shortcomings that may be the source of the differences in simulated versus observed biomass at HBEF. In a recent study, Battles et al. (2014) showed a reversal of forest decline at HBEF following the addition of Ca^2+^ silicate, which resulted in higher photosynthetic area and above ground primary production. These findings potentially open the way for possible improvements to the model.

### 4.3 Ground Vegetation Composition

The calibrated vegetation responses produced a good fit between the observed and modeled occupancies at the three sites. The calibration procedure required limited modifications to the original expert parameterization (see the “Model Calibration Procedure” section above for a description of the modified parameters), with a notable increase in performance. The implications of this are twofold. First, it appears that the model is reasonable at projecting the relative abundances of the 45 species for which it was calibrated, and that performance is consistent across the three sites with a single common calibrated table. However, the sensitivity of the performance to calibrated versus non-calibrated data suggests that the model should not be used with non-calibrated species, and that greater ground-truthing with compositional data is needed. Furthermore, without temporal data that characterizes changes in plant cover, it is unclear whether the good fit with calibrated species will hold over time. However, based on the concept of a species’ fundamental niche, which is independent of local conditions and captured in the model, the correspondence is not hypothesized to disappear. Further tests of the calibrated species responses on independent data sources are needed to test this hypothesis.

The calibration of the plant niches was successful over the spread of community structures at the three sites. The sites were distinctly different in that the understory at HBEF was clearly dominated by two main species, while plant dominance was more gradually distributed at EB and no plant cover exceeded 20% at WB. These results may offer a reason to strengthen confidence in the modeling of plant community composition, particularly when combining this study with other findings in, for example, Dirnbök et al. (2017). Moreover, it remains important to note that even with satisfactory confidence, the use of a single metric for plant community composition may limit the efficacy of models to inform about the need for protection from anthropogenic changes. Other indicators and metrics are for example presented and discussed in Rowe et al. (2017).

### 4.4 Implication for Integrated Forest Ecosystem Modeling

Dynamic modeling in general, and dynamic ecosystem modeling in particular, faces the continuous challenge of trying to balance good performance with limited calibration. The reason for this is by trying to create simulation tools that are able to reproduce ecosystem behavior from our understanding of core processes, rather than heavily forcing those tools to mimic observations through opaque calibration of parameters. Only then are we able to have confidence in model projections of highly dynamic futures, where multiple drivers change simultaneously on top of the ecosystems’ internal feedbacks. Yet, it is not uncommon for models to fall in the “right behavior for the wrong reason” dilemma. This study can be seen as an example of the potential of integrated monitoring to help solve that dilemma. By providing information on most key ecosystem components, empirical data of the quality found at the sites simulated here forces the models to get each component right.

The interpretation of the empirical observations is obviously enhanced by the availability of long time series of data. This is also enhanced by integrated modeling that provides a platform to test how different process descriptions fit together, and if congruous can reproduce the overall observed behavior. It is maybe not a surprise that many terrestrial ecosystem models originated or were refined based on the data at HBEF and BBWM (see for example McDonnell et al. 2018a; McDonnell et al. 2018b; Gbondo-Tugbawa et al. 2001; Aber and Federer 1992; Cosby et al. 1996; Pourmokhtarian et al. 2017). This study confirms the importance and efficacy of combining integrated long-term ecosystem monitoring and dynamic modeling, and can only encourage more such efforts, particularly in view of the simultaneous and increasingly major environmental changes affecting our ecosystems.

## 5 Conclusions

This study shows that although the modeling exercise made use of multiple data sources, the data were robust and coherent. It is this coherence in input data that made the modeling and model evaluation possible and successful with limited calibration. The biogeochemical model performed reasonably well with minimal calibration, supporting the prospect of using the model on sites where data scarcity may limit the calibration.

The comparisons with field data (particularly site observations and soil solution measurements) provided a unique and powerful opportunity to test the concepts and assumptions of the ForSAFE-Veg model. Although the annual ranges of the modeled data fitted well with the observations, the seasonal variability produced by the model exceeded the observed fluctuations. This sheds valuable light on the shortcoming of the biogeochemical component of the model, where seasonality is imbedded in virtually all processes. Thus, it appears that the model is well suited to simulate dynamics that occur over yearly or multi-year periods (e.g., changes in soil biogeochemistry, and plant community composition to a lesser extent), and poorly suited at present to simulate sub-annual dynamics (e.g., instantaneous leaching rates, intra-annual dynamics of plant competition such as shading).

The model performed acceptably well at sites undergoing different transient changes, such as biomass clearing, different climatic conditions, and different soil amendment levels. This strengthens confidence in the model to simulate plant and biogeochemical response to simultaneous climatic and deposition changes. Unlike the biogeochemical component, the vegetation composition module required calibration. Although the calibration was limited, its impact on model performance was substantial. This emphasizes both the sensitivity of the plant community composition to the niches and the reliability of the expert opinions on which the original parameterization is based.

## Figures and Tables

**Table 1:** Observed and modeled relative cover distributions of forest floor plants at the Hubbard Brook Experimental Forest (HBEF) and East Bear (EB) and West Bear (WB) sites. Values for the EB and WB watersheds are from the year 1997, and from the year 2013 for HBEF. The Blind and Cal. columns refer, respectively, to the simulated covers using the raw expert parametrization of the plant’s ecological niches and traits, and the calibrated parametrization. The values are rounded to the second decimal (showing a resolution down to 1%)

	HBEF			EB			WB		
	Observed	Modeled		Observed	Modeled		Observed	Modeled	
Plant		Blind	Cal.		Blind	Cal.		Blind	Cal.

*Abies balsamea*				0.00	0.14	0.01	0.00	0.01	0.01
*Acer pensylvanicum*	0.01	0.04	0.05	0.07	0.01	0.02	0.14	0.08	0.15
*Acer rubrum*				0.02	0.14	0.02	0.03	0.10	0.04
*Acer saccharum*	0.01	0.00	0.02	0.03	0.00	0.01	0.14	0.03	0.15
*Aralia nudicaulis*				0.03	0.00	0.04	0.03	0.01	0.01
*Arisaema triphyllum*				0.00	0.00	0.00			
*Betula alleghaniensis*	0.02	0.00	0.03	0.13	0.14	0.12	0.04	0.01	0.01
*Clintonia borealis*	0.04	0.04	0.04				0.00	0.02	0.02
*Coptis trifolia*				0.01	0.00	0.00	0.00	0.00	0.00
*Cornus alternifolia*				0.00	0.00	0.00	0.00	0.00	0.00
*Cornus canadensis*				0.01	0.03	0.02	0.00	0.00	0.00
*Dennstaedtia punctilobula*	0.00	0.68	0.03	0.00	0.02	0.02	0.00	0.04	0.05
*Diervilla lonicera*				0.00	0.00	0.00			
*Dryopteris campyloptera*				0.21	0.00	0.15	0.16	0.00	0.19
*Dryopteris intermedia*	0.32	0.00	0.33						
*Fagus grandifolia*	0.07	0.00	0.05	0.25	0.01	0.22	0.18	0.08	0.18
*Fragaria virginiana*				0.00	0.01	0.01			
*Fraxinus americana*				0.00	0.01	0.01	0.00	0.04	0.00
*Gymnocarpium dryopteris*				0.00	0.00	0.00	0.03	0.00	0.00
*Huperzia lucidula*	0.10	0.00	0.06						
*Lonicera canadensis*				0.00	0.00	0.00	0.00	0.00	0.00
*Maianthemum canadense*	0.03	0.00	0.00	0.02	0.00	0.00	0.01	0.00	0.00
*Maianthemum racemosum*	0.01	0.00	0.00						
*Medeola virginiana*	0.00	0.00	0.00	0.00	0.00	0.00	0.00	0.00	0.00
*Monotropa uniflora*	0.00	0.00	0.00						
*Oclemena acuminata*	0.01	0.00	0.00						
*Osmunda claytoniana*				0.05	0.00	0.03	0.00	0.19	0.01
*Oxalis montana*	0.02	0.00	0.01						
*Phegopteris connectilis*				0.00	0.00	0.00			
*Picea rubens*				0.07	0.10	0.08	0.04	0.01	0.01
*Polygonatum pubescens*							0.00	0.00	0.00
*Polygonum convolvulus*				0.00	0.00	0.00	0.00	0.00	0.00
*Polystichum acrostichoides*							0.00	0.00	0.00
*Prunus serotina*				0.00	0.00	0.00	0.00	0.06	0.00
*Quercus rubra*							0.00	0.06	0.00
*Ribes glandulosum*				0.00	0.00	0.01			
*Sorbus americana*				0.00	0.10	0.01	0.00	0.05	0.00
*Phegopteris connectilis*							0.00	0.00	0.00
*Thelypteris noveboracensis*				0.00	0.05	0.00	0.03	0.00	0.00
*Trientalis borealis*	0.00	0.00	0.00	0.02	0.00	0.00	0.01	0.00	0.00
*Trillium spp*	0.00	0.00	0.00				0.00	0.00	0.00
*Tsuga canadensis*				0.02	0.14	0.01			
*Uvularia sessilifolia*	0.04	0.00	0.05	0.05	0.00	0.01	0.07	0.02	0.04
*Viburnum acerifolium*	0.29	0.25	0.34	0.00	0.00	0.00	0.00	0.00	0.00
*Viburnum lantanoides*				0.00	0.12	0.02	0.07	0.14	0.07
*Viola rotundifolia*	0.00	0.00	0.00						

## Appendix 1

This section provides a detailed description of the model input data introduced in the “Method” section of the main paper.

### Soil and mineralogy data

Soil data for Hubbard Brook Experimental Forest (HBEF) were from soil samples (Bhs, Bs1, Bs2, Bs3, and Cd horizons) collected in 1997 from a soil profile pit in the plot west of W6 (Bailey et al. 2004, personal communication), unless otherwise specified in Table 2. Fine earth (< 2 mm) bulk densities and hydrological properties were determined with equations from Balland et al. (2008), using particle size distribution and loss on ignition from the soil samples (J. Aherne, personal communication). At HBEF, the calculated values of fine earth bulk density compared well with the field measurements from Johnson et al. (1991a), and the calculated hydrological parameters related well to the values reported in Federer (1992). The referenced values in Johnson et al. (1991a) and Federer (1992) were not used because not all modeled soil layers were represented. Soil surface areas were calculated by applying particle size distributions to the equations outlined by Phelan et al. (2014).

**Table 2:** Soil parameters at Hubbard Brook Experimental Forest (HBEF)

Soil parameter	Ref.	Unit	Soil horizon							
			O	A	E	Bhs	Bs1	Bs2	Bs3	Cd

Horizon thickness	1	M	0.07	0.07	0.03	0.04	0.1	0.1	0.55	0.11
Bulk density	2	kg/m^3^	177.0	351.6	997.3	666.3	658.7	795.3	874.4	1100.3
Soil surface area	3	10^6^ m^2^/m^3^	0	0.13	0.59	0.22	0.32	0.48	0.72	1.10
Kgibb	4	log_10_(m^6^/eq^2^)	6.5	6.5	6.5	7.6	7.6	8.6	8.6	9.2
Cation exchange capacity	5	meq/kg	175	72.5	25.4	147.2	104.3	54.6	38.8	13.9
Base saturation	5	Fraction	0.5	0.64	0.16	0.05	0.03	0.02	0.04	0.07
Field capacity	2	m^3^/m^3^	0.79	0.47	0.12	0.2	0.23	0.17	0.17	0.14
Wilting point	2	m^3^/m^3^	0.43	0.21	0.04	0.08	0.09	0.06	0.06	0.04
Field saturation	2	m^3^/m^3^	0.87	0.88	0.61	0.72	0.72	0.67	0.65	0.58
Fine root distribution	6	% of total	26	24	10	8	16	12	4	0
Minerals	7	% of total								
K-feldspar			–	15.70	15.70	13.82	6.31	7.11	9.37	12.10
Muscovite				0.52	0.52	3.85	13.43	13.13	10.37	8.51
Hornblende				1.50	1.50	1.85	1.82	3.09	3.16	3.16
Plagioclase				17.29	17.29	18.83	19.66	22.07	20.35	22.67
Fe-chlorite				0.79	0.79	6.08	3.69	2.21	2.09	2.02
Mg-vermiculite				0.17	0.17	0.26	0.23	0.32	0.35	0.41
Apatite				0.15	0.15	0.28	0.32	0.41	0.38	0.45
Kaolinite				0.30	0.30	2.25	5.31	5.93	6.06	4.97
Calcite				0.35	0.35	0.35	0.35	0.37	0.30	0.43

(1) Bailey (personal communication), Federer (1992)

(2) Calculated according to Balland et al. (2008) from soil texture (J. Aherne, personal communication)

(3) Calculated according to Phelan et al. (2014) from soil texture (J. Aherne, personal communication)

(4) Warfvinge and Sverdrup (1994)

(5) Bailey (personal communication), Johnson et al. (1991b)

(6) Yanai et al. (2006)

(7) J. Aherne (personal communication)

The proportions (% weight) and stoichiometry of each mineral (given in Table 3) were determined through a combination of qualitative mineralogy and total oxide analysis results (J. Aherne, personal communication) and the Analysis to Mineralogy model (A2M version 1.3, Posch and Kurz 2007).

**Table 3:** Stoichiometries of mineral classes used for Hubbard Brook Experimental Forest (HBEF)

Mineral	Horizon	Stoichiometry (elemental content)									
		Si	Al	Fe	Mn	Mg	Ca	K	Na	P	Ti

Apatite*	A/E, B, C	0.00	0.00	0.00	0.00	0.00	5.00	0.00	0.00	3.00	0.00
Calcite	A/E	0.00	0.00	0.00	0.00	0.35	1.65	0.00	0.00	0.00	0.00
	B	0.00	0.00	0.00	0.00	0.50	1.50	0.00	0.00	0.00	0.00
	C	0.00	0.00	0.00	0.00	0.56	1.44	0.00	0.00	0.00	0.00
Fe-chlorite	A/E	3.00	2.00	3.82	0.46	0.72	0.00	0.00	0.00	0.00	0.00
	B	3.00	2.00	4.34	0.15	0.51	0.00	0.00	0.00	0.00	0.00
	C	3.00	2.00	4.06	0.28	0.67	0.00	0.00	0.00	0.00	0.00
Hornblende	A/E	8.00	0.00	4.31	0.00	0.69	2.00	0.00	0.00	0.00	0.00
	B	8.00	0.00	4.49	0.00	0.51	2.00	0.00	0.00	0.00	0.00
	C	8.00	0.00	4.25	0.00	0.75	2.00	0.00	0.00	0.00	0.00
K-feldspar	A/E, B, C	3.00	1.00	0.00	0.00	0.00	0.00	1.00	0.00	0.00	0.00
Mg-vermiculite	A/E, B, C	5.50	2.50	1.00	0.00	5.19	0.13	0.39	0.21	0.00	0.00
Muscovite	A/E, B, C	3.00	3.00	0.00	0.00	0.00	0.00	1.00	0.00	0.00	0.00
Plagioclase	A/E	2.97	1.03	0.00	0.00	0.00	0.03	0.00	0.97	0.00	0.00
	B	2.96	1.04	0.00	0.00	0.00	0.04	0.00	0.96	0.00	0.00
	C	2.96	1.04	0.00	0.00	0.00	0.04	0.00	0.96	0.00	0.00

*Si* silicon, *Al* aluminum, *Fe* Iron, *Mg* magnesium, *Ca* calcium, *K* potassium, *Na* sodium, *P* phosphorus, *Ti* titanium

* Apatite was not identified by the X-ray diffraction but added to account for P in the total elemental analysis

At East Bear (EB) and West Bear (WB) watersheds in Bear Brook Watershed Maine (BBWM), soil samples collected in 2010 from three hardwood forest locations in EB served as the source of horizon thickness, soil particle size distributions, and soil mineralogy estimates. Samples from EB were selected to represent select soil parameters for both watersheds because the watersheds are small, are in close proximity, and contain soils developed from the same parent material (well-mixed glacial till) (I. Fernandez, personal communication). Similar to HBEF, fine earth bulk densities were estimated using the Balland et al. (2008) equation and particle size distributions conducted on the three soil samples from EB (J. Aherne, personal communication) and loss on ignition estimates specific to each of the two plots (SanClements et al. 2010). Soil surface areas and mineralogy were determined using the same methods outlined for HBEF. Tables 4 and 5 present the soil parameters used in the ForSAFE simulations for EB and WB, respectively. Table 6 presents the mineral contents data used for both sites.

**Table 4:** Soil parameters used in the ForSAFE simulation for East Bear (EB) watershed

Soil parameter	Ref.	Unit	Soil horizon				
			O	B1	B2	B3	C

Thickness	1	M	0.055	0.05	0.25	0.14	0.58
Bulk density	2	kg/m^3^	217.9	587.5	675.0	794.0	1017.7
Area	3	m^2^/m^3^	64,953	507,947	639,356	903,193	1,377,622
Kgibb	4	log_10_(m^6^/eq^2^)	6.5	7.6	8.6	8.6	9.5
CEC	1	meq/kg	230.0	70.0	57.0	27.0	33.0
Base saturation	1	Fraction	58	9	7.9	8.5	16.2
Fc	2	m^3^/m^3^	0.69	0.36	0.32	0.29	0.26
Wp	2	m^3^/m^3^	0.35	0.12	0.10	0.09	0.06
Fs	2	m^3^/m^3^	0.86	0.74	0.71	0.68	0.60
Roots	5	Fraction of total	0.13	0.13	0.29	0.35	0.1
Minerals	6	% of total					
K-feldspar			4.47	4.47	4.47	4.47	7.26
Muscovite			14.95	14.95	14.95	14.95	10.36
Hornblende			2.70	2.70	2.70	2.70	2.54
Plagioclase			14.46	14.46	14.46	14.46	15.19
Fe-chlorite			8.48	8.48	8.48	8.48	7.44
Mg-vermiculite			10.36	10.36	10.36	10.36	10.32
Apatite			0.32	0.32	0.32	0.32	0.23
Calcite			0.00	0.00	0.00	0.00	0.14

(1) SanClements et al. (2010)

(2) Calculated according to Baland et al. (2008) from soil texture (J. Aherne, personal communication)

(3) Calculated according to Phelan et al. (2014) from soil texture (J. Aherne, personal communication)

(4) Warfvinge and Sverdrup (1994)

(5) Yanai et al. (2006)

(6) J. Aherne (personal communication)

**Table 5:** Soil parameters used in the simulations for West Bear (WB) watershed (only parameters differing from EB are shown)

Soil parameter	Ref.	Unit	Soil horizon				
			O	B1	B2	B3	C

Bulk density	1	kg/m^3^	190.2	491.3	701.3	830.6	1017.7
Area	2	m^2^/m^3^	34,591	390,111	672,417	955,662	1,377,622
CEC	3	meq/kg	220.0	100.0	41.0	28.0	15.0
Base saturation	3	Fraction	22	7	7.2	6.7	7.1
Fc	1	m^3^/m^3^	0.73	0.41	0.31	0.28	0.26
Wp	1	m^3^/m^3^	0.40	0.15	0.10	0.08	0.06
Fs	1	m^3^/m^3^	0.87	0.77	0.71	0.66	0.60

(1) Calculated according to Baland et al. (2008) from soil texture (J. Aherne, personal communication)

(2) Calculated according to Phelan et al. (2014) from soil texture (J. Aherne, personal communication)

(3) SanClements et al. (2010)

**Table 6:** Elemental contents of the mineral reported at East Bear (EB) and West Bear (WB) watersheds

Mineral class	Horizon	Elemental content									
		Si	Al	Fe	Mn	Mg	Ca	K	Na	P	Ti

Apatite	B, C	0.00	0.00	0.00	0.00	0.00	5.00	0.00	0.00	3.00	0.00
Calcite	C	0.00	0.00	0.00	0.00	0.00	1.00	0.00	0.00	0.00	0.00
Fe-chlorite	B	3.00	2.00	4.64	0.04	0.31	0.00	0.00	0.00	0.00	0.00
	C	3.00	2.00	4.56	0.04	0.39	0.00	0.00	0.00	0.00	0.00
Hornblende	B	8.00	0.00	3.01	0.00	1.99	2.00	0.00	0.00	0.00	0.00
	C	8.00	0.00	3.13	0.00	1.87	2.00	0.00	0.00	0.00	0.00
K-feldspar	B, C	3.00	1.00	0.00	0.00	0.00	0.00	1.00	0.00	0.00	0.00
Mg-vermiculite	B	4.13	1.72	0.09	0.00	0.77	0.10	0.04	0.17	0.00	0.00
	C	4.12	1.72	0.08	0.00	0.74	0.08	0.03	0.19	0.00	0.00
Muscovite	B	3.14	2.86	0.00	0.00	0.00	0.00	0.86	0.00	0.00	0.00
	C	3.12	2.88	0.00	0.00	0.00	0.00	0.88	0.00	0.00	0.00
Plagioclase	B	2.94	1.06	0.00	0.00	0.00	0.06	0.00	0.94	0.00	0.00
	C	2.96	1.04	0.00	0.00	0.00	0.04	0.00	0.96	0.00	0.00

### Tree Tissue Nutrient Contents

Tree species compositions at the three sites were determined using the measurements of diameters at breast height converted into tree biomass using the Hubbard Brook singletree biomass program (http,//www.hubbardbrook.org/w6_tour/biomass-stop/single-tree-biomass.htm), which is based on the work of Whitacker and others (1974). Using the tissue nutrient contents of each of the tree species together with their respective fractions of the total biomass, it was possible to derive foliar, wood, and root nutrient contents for the three forest stands (Tables 7 and 8).

**Table 7:** Forest stand tissue nutrient concentrations at Hubbard Brook Experimental Forest (HBEF)

Tissue type	Nutrient contents (mg/g)				Sources
	N	Mg	Ca	K	

Foliage*	24–30	1.5–2.25	7–10.5	8–16	Pham et al. (1978); Ollinger et al. (2002); Halman et al. (2008); Wicklein et al. (2012); Park and Yanai (2009)
Wood	1.5	0.12	0.5	0.5	Park and Yanai (2009)
Fine roots	1.2	0.7	3	2.5	Park and Yanai (2009); Likens et al. (1994)

* The model internally calculates the foliar nutrient content within the given range depending on the availability of the nutrients

**Table 8:** Forest stand tissue nutrient contents weighted by dominant tree abundance at Bear Brook Watershed Maine (BBWM)

Tissue type	Nutrient contents (mg/g)				Sources
	N	Mg	Ca	K	

Foliage	20	1.2	4.0	7.0	Pham et al. (1978); Ollinger et al. (2002); Halman et al. (2008); Wicklein et al. (2012); Park and Yanai (2009)
Wood	1.5	0.10	0.80	0.45	Park and Yanai (2009)
Fine roots	8.0	0.50	2.0	0.5	Park and Yanai (2009); Likens et al. (1994)

## Appendix 2

This appendix presents the plant niches database (Table 10) used in the simulations, as well as a description of the niches classes.

To facilitate the parameterization of the ecological niches, plant optima were defined in classes (Table 9) corresponding to specific field values (Table 10)

**Table 9:** Trait and niche classes and their corresponding values

Plant trait	Veg designation	Veg class	Description	Corresponding values	Unit

Physiological traits					
Colonization pace	*τ*_in_	1	Fast colonizers	*τ*_in_ <2	years
		2	Reasonably fast colonizers	2 ≤ *τ*_in_ <5	
		3	Slow colonizers	5 ≤ *τ*_in_ < 10	
		4	Very slow colonizer	*τ*_in_ ≥ 10	
Retreat pace	*τ*_out_	1	Easily eradicated, very sensitive plants	*τ*_out_ ≤ 2	years
		2	Sensitive plant	2 < *τ*_out_ ≤ 5	
		3	Relatively hardy plants	5 < *τ*_out_ ≤ 10	
		4	Tenacious plant	*τ*_out_ > 10	
Rooting depth	Roots	1	Plants with no roots	Root depth = 0	cm
		2	Shallow-rooted plants	Root depth ≤ 15	
		3	Plants with intermediate root depth	15 < root depth ≤ 45	
		4	Deep-rooted plants	Root depth > 45	
Shading height	Shading height	–			m
Palatability**	Palatability	1	Toxic	–	–
		2	Avoided, but eaten in times of shortage	–	
		3	Acceptable, eaten when better food is scarce	–	
		4	Good, generally browsed on	–	
		5	Very sought for, heavily grazed on	–	
Climatic niches Temperature window (ranges)	*T*_min_/*T*_max_	–	Window of temperatures within which a plant can establish	*T*_min_ ≤ plant *T* niche ≤ *T*_max_	°C
Shade tolerance/light demand (optima)	Light	1 2 3 4 5	Very shade tolerant Shade tolerant Half-shade Light demanding Very light demanding,	0.03 0.065 0.20 0.70 0.95	Fraction of ambient light
Water requirements (optima)	Moisture	1	High drought tolerance	0.05	Unitless (fraction above wilting point*)
		2	Medium drought tolerance	0.1	
		3	Plants thrive under well-drained but not dry conditions	0.3	
		4	Plants thrive under moist conditions	0.6	
		5	Plants live under water saturated conditions	1.0	
Chemical niches					
Nitrogen	N	1	Very low N demand	0.1	mg_N_/l
		2	Low N demand	0.4	
		3	Moderate N demand	0.8	
		4	High N demand	1.5	
		5	Very high N demand	2.0	
pH	pH	1	Acidic < 4		
		2	Moderately acidic 4–5.5		
		3	Neutral 5.5–6.5		
		4	Basic > 6.5		

* This is the water saturation fraction above wilting point = (*θ* − wp)/(fs − wp), where *θ* is the average yearly volumetric water content (m^3^ /m^3^ ), wp is the soil’s wilting point (m^3^ /m^3^ ), and fs is the soil’s field saturation (m^3^ /m^3^ )

** Palatability was not activated in the present simulations

For drivers defined with optima (light demand, water requirements, N and pH), the plants’ niches definitions also contain indicators of variability (used in Eq. 1 in the main paper). The light variability indicates the number of light classes, including the optimal, that a plant tolerates. Dry and wet tolerance indicators denote the number of classes below or above the optimal that a plant can still tolerate. Nitrogen variability indicates the number of N classes above the optimal that a plant tolerates. Acid and basic tolerance indicators represent the number of pH classes below or above the optimal pH that a plant tolerates.

**Table 10:** Plant niches and traits used in the plant community composition module Veg

	Physiological traits					Climatic response			
Plant	*τ*_in_	*τ*_out_	Root	Shading height	Palatability	*T*_min_	*T*_max_	Light class	Light variability

*Abies balsamea*	3	4	4	6	4	3	12.5	5	III
*Acer pensylvanicum*	2	4	4	6	4.5	3.9	15.6	3.5	II
*Acer rubrum*	2	4	4	6	5	3	24.4	4.5	IV
*Acer saccharum*	2	4	4	6	5	3	19.6	3.5	III
*Aralia nudicaulis*	2	3	3	3.5	4	2.4	15.2	4	II
*Arisaema triphyllum*	3	3	3	3	2	3	23.7	2	II
*Betula alleghaniensis*	2	4	4	6	5	3	14.9	5	III
*Clintonia borealis*	4	2	2	2	2	3	13.8	3	III
*Coptis trifolia*	2	2	2	1.5	1	3	12.7	2	II
*Cornus alternifolia*	2	3	4	6	5	3.5	19.7	3	II
*Cornus canadensis*	2	2	3	2	2.5	1.4	13.2	3	III
*Dennstaedtia punctilobula*	1	2	3	4	1	3.9	17.2	4	III
*Diervilla lonicera*	2	3	4	4.5	2.5	3	15.7	4	III
*Dryopteris campyloptera*	2	2	2	4	2.5	4	15.7	3	III
*Dryopteris intermedia*	2	2	2	5	2.5	3	15.9	2.5	III
*Fagus grandifolia*	3	4	3	6	2.5	3.9	20.7	3.5	IV
*Fragaria virginiana*	1	2	2	2	3	0.7	20.1	5	III
*Fraxinus americana*	2	4	4	6	4.5	3.9	21.8	5	III
*Gymnocarpium dryopteris*	2	2	2	2	1	1.5	13.4	2	II
*Huperzia lucidula*	2	2	2	3	1	3	16.3	2	III
*Lonicera canadensis*	2	3	4	5	4.5	3	13.1	2.5	II
*Maianthemum canadense*	1	1	2	2	4.5	3	15.1	2.5	II
*Maianthemum racemosum*	1	1	2	3	4	1.2	20.1	2	II
*Medeola virginiana*	2	1	2	3	4	3.9	19.7	2.5	II
*Oclemena acuminata*	1	2	3	3	2	3.9	14.2	4.5	II
*Osmunda claytoniana*	2	2	3	5	2	3	16	1.5	III
*Oxalis montana*	1	2	2	1.5	2	3	15.6	3	II
*Phegopteris connectilis*	2	3	2	2.5	1	3	14.3	2	II
*Picea rubens*	3	4	4	6	2	3.9	13.4	5	III
*Polygonatum pubescens*	1	2	2	3	1	3	14.5	2.5	II
*Polygonum convolvulus*	1	2	2	3.5	2	2.4	23.5	4	III
*Polystichum acrostichoides*	1	2	2	2.5	1	3.9	21.8	2	II
*Prunus serotina*	2	4	4	6	2	3.9	22.9	5	II
*Prunus virginiana*	2	2	2	3	2	5.5	21.8	3	III
*Quercus rubra*	3	4	4	6	4	3	19.6	5	II
*Ribes glandulosum*	2	3	4	3.5	4	3	13.8	3	II
*Sorbus americana*	2	3	4	6	5	3	15.8	5	II
*Thelypteris noveboracensis*	2	3	3	3	1	3.9	19.8	3.5	III
*Trientalis borealis*	1	2	2	2	2	3	15.9	2	II
*Trillium spp*	1	3	3	3	4.5	3.9	15.8	2	II
*Tsuga canadensis*	3	4	4	6	3	4.1	17.4	5	V
*Uvularia sessilifolia*	1	2	2	2.5	3.5	3	19.6	3	III
*Viburnum acerifolium*	2	4	3	5	4	4.2	20.2	3	II
*Viburnum lantanoides*	2	4	4	5	5	3.9	14	2	III
*Viola rotundifolia*	1	2	2	1	2	4.1	15.5	2.5	II

	Climatic response				Soil chemical niches				
Plant	Moisture class		Dry tolerance	Wet tolerance	N class	N variability	pH Class	Acid tolerance	Basic tolerance

*Abies balsamea*	3		II	III	2	II	1.5	II	III
*Acer pensylvanicum*	3		II	II	2	III	2	II	II
*Acer rubrum*	3		III	IV	2	IV	2	III	II
*Acer saccharum*	3.5		II	II	1.5	IV	2	III	III
*Aralia nudicaulis*	3		II	II	1.5	IV	2	III	III
*Arisaema triphyllum*	3.5		I	II	2.5	IV	2.5	II	II
*Betula alleghaniensis*	3		II	III	1.5	III	2	II	II
*Clintonia borealis*	3		I	II	1.5	IV	1.5	II	II
*Coptis trifolia*	4		II	II	1.5	IV	1.5	II	II
*Cornus alternifolia*	3		II	II	2.5	III	3	II	III
*Cornus canadensis*	3		III	III	1	II	2.5	II	I
*Dennstaedtia punctilobula*	2		III	III	2	IV	2	III	I
*Diervilla lonicera*	2.5		II	II	1.5	III	2	II	II
*Dryopteris campyloptera*	3		II	II	2	IV	1.5	III	II
*Dryopteris intermedia*	3		II	II	2	IV	2	III	II
*Fagus grandifolia*	3		II	II	2	III	2	II	II

	Climatic response				Soil chemical niches					
Plant	Moisture class		Dry tolerance	Wet tolerance	N class	N variability	pH Class	Acid tolerance	Basic tolerance

*Fragaria virginiana*	3		II	II	1.5	V	2.5	III	II
*Fraxinus americana*	3		II	II	2.5	V	2.5	II	III
*Gymnocarpium dryopteris*	3.5		II	II	2	II	2	II	III
*Huperzia lucidula*	3		II	II	1.5	IV	2	II	II
*Lonicera canadensis*	3.5		I	II	2	II	2	II	II
*Maianthemum canadense*	3		II	II	1.5	III	2	II	III
*Maianthemum racemosum*	3		II	II	2	III	2.5	II	III
*Medeola virginiana*	3		II	II	2	II	2	II	II
*Oclemena acuminata*	3		II	II	1.5	III	2	II	II
*Osmunda claytoniana*	3		II	II	2	V	2	II	II
*Oxalis montana*	3		II	III	1.5	III	2	III	III
*Phegopteris connectilis*	3.5		II	II	2	II	2	II	III
*Picea rubens*	3		II	III	1.5	II	2	I	III
*Polygonatum pubescens*	3		II	V	2	III	2.5	II	III
*Polygonum convolvulus*	2.5		II	II	2.5	III	2	II	III
*Polystichum acrostichoides*	3		II	V	2	V	2.5	I	III
*Prunus serotina*	3		III	II	2	V	1.5	II	II
*Prunus virginiana*	2		II	I	1	II	1.5	II	II
*Quercus rubra*	3		III	II	1.5	IV	2	II	II
*Ribes glandulosum*	3.5		II	III	2	III	2	II	III
*Sorbus americana*	3		II	III	2	IV	2	II	II
*Thelypteris noveboracensis*	2.5		III	II	1	V	2	II	I
*Trientalis borealis*	3		II	II	1.5	III	1.5	I	III
*Trillium spp*	3		II	II	2	III	2	II	II
*Tsuga canadensis*	3		IV	IV	2	II	1.5	II	III
*Uvularia sessilifolia*	3		II	II	3	IV	2	II	II
*Viburnum acerifolium*	2.5		II	II	2	II	2	II	II
*Viburnum lantanoides*	3		II	II	2	III	2	II	II
*Viola rotundifolia*	3		II	II	1.5	IV	2	I	II
